# Safety and efficacy of zuranolone in Japanese adults with major depressive disorder: An open‐label, repeated‐treatment part of a Phase 3 clinical trial

**DOI:** 10.1002/pcn5.70302

**Published:** 2026-02-19

**Authors:** Masaki Kato, Kazuyuki Nakagome, Takamichi Baba, Takuhiro Sonoyama, Hiroki Fukuju, Ryosuke Shimizu, Juan Carlos Gomez, Tomoko Motomiya, Takeshi Inoue

**Affiliations:** ^1^ Department of Neuropsychiatry Kansai Medical University Osaka Japan; ^2^ Department of Psychiatry National Center of Neurology and Psychiatry Tokyo Japan; ^3^ Drug Development and Regulatory Science Division Shionogi & Co., Ltd. Osaka Japan; ^4^ Drug Development and Regulatory Science Division Shionogi B.V. London UK; ^5^ Department of Psychiatry Tokyo Medical University Tokyo Japan; ^6^ Department of Psychiatry Sapporo Hanazono Hospital Hokkaido Japan

**Keywords:** depressive disorder, Japan, Phase 3, safety, zuranolone

## Abstract

**Aim:**

To evaluate the safety and tolerability of repeating 14‐day treatment with zuranolone (30 mg) followed by a 6‐week follow‐up period (one treatment cycle) for a maximum of six treatment cycles in Japanese participants with major depressive disorder.

**Methods:**

This multicenter Phase 3 study was conducted in two parts (70 sites; Japan). Part B was an open‐label study for participants who completed the initial double‐blind study (Part A; zuranolone vs. placebo) in which all eligible participants received zuranolone regardless of their assignments in Part A. Endpoints included treatment‐emergent adverse events (TEAEs) and efficacy using the 17‐item Hamilton Depression Rating Scale (HAMD‐17).

**Results:**

A total of 271, 213, 170, 147, 126, and 99 participants were evaluated in Cycles 1–6, respectively. TEAEs occurring in ≥5% of participants were somnolence and dizziness during the treatment period and nasopharyngitis during the follow‐up period, and no new safety signals were observed with increased numbers of treatment cycles. Additionally, no TEAEs suggestive of drug dependence or withdrawal symptoms were identified. The HAMD‐17 total score decreased by 8 and 15 days after the initiation of zuranolone in all six cycles, and there were no meaningful differences in the HAMD‐17 total score reduction across treatment cycles.

**Conclusion:**

In this open‐label part of the Phase 3 study in Japan, zuranolone decreased the HAMD‐17 total scores from baseline over 15 days during repeated treatment cycles in patients with major depressive disorder, without any new safety signals.

## INTRODUCTION

Major depressive disorder (MDD) is a clinical progressive disorder^1^ that can lead to treatment‐resistant depression and increased risk of suicide.^2^ The time to response of currently available antidepressants for the treatment of MDD can range from weeks to months for some patients,^3^, ^4^, ^5^, ^6^, ^7^ suggesting an unmet need for fast symptom improvement.^8^

Zuranolone (SAGE‐217 in the United States and S‐812217 in Japan), an oral synthetic neuroactive steroid (gamma‐aminobutyric acid‐A receptor–positive modulator) with a novel mechanism of action, has been approved by the United States Food and Drug Administration for the treatment of postpartum depression in adults.^9^ Zuranolone has a molecular pharmacology profile similar to that of the endogenous neuroactive steroid allopregnanolone^10^ and has been evaluated in several clinical trials.^11^, ^12^, ^13^, ^14^, ^15^, ^16^, ^17^ Zuranolone has been shown to ameliorate depressive symptoms as early as Day 3 of treatment.^15^, ^18^, ^19^, ^20^

In a Phase 2 dose‐finding placebo‐controlled study in Japan, oral zuranolone showed improvements in depressive symptoms as assessed using the 17‐item Hamilton Depression Rating Scale (HAMD‐17) total score change from baseline over 14 days in Japanese patients with MDD, with an acceptable safety profile and tolerability. The difference in the adjusted mean HAMD‐17 total score between the zuranolone 30‐mg and placebo groups and between the zuranolone 20‐mg and placebo groups was statistically significant on Day 15.^19^

Based on the results of the Phase 2 dose‐finding study, the 30‐mg dose was selected for this multicenter Phase 3 study in Japanese patients with MDD, which was conducted in two parts. Part A was an 8‐week study comprising a 2‐week treatment period after screening and a 6‐week follow‐up period, in which zuranolone improved depressive symptoms on Day 15 compared with placebo, as assessed by the change from baseline in the HAMD‐17 total score.^21^

Part B was an open‐label extension study conducted in participants who had completed Part A, in which they were followed up and re‐treated as required. The aim of Part B was to evaluate the safety and tolerability of repeating the 14‐day treatment with zuranolone, followed by a 6‐week follow‐up (observation/follow‐up) period for a maximum of six treatment cycles over a year in Japanese participants with MDD. Herein, we present the results from Part B.

## METHODS

### Study design

This multicenter Phase 3 study (jRCT2031210577) was conducted in two parts in 70 medical institutions across Japan. Part A was a randomized, double‐blind, placebo‐controlled, parallel‐group study in participants with MDD.^21^ Part B was an open‐label study conducted in participants who completed Part A irrespective of treatment received in Part A (zuranolone 30 mg once daily or placebo), in which participants were re‐treated with zuranolone 30 mg once daily as required (zuranolone/zuranolone group and placebo/zuranolone group) if they met the re‐treatment criteria: HAMD‐17 total score ≥ 14 and depressive episodes persisting for ≥2 weeks (based on Diagnostic and Statistical Manual of Mental Disorders, 5th edition [DSM‐5]). The treatment assignment during Part A was blinded. Currently available data suggest that zuranolone 30 mg has an acceptable safety profile and tolerability^21^; therefore, it was not considered necessary to establish dose‐reduction criteria in this study.

Participants who consented to participate in Part B between Visit 9 and Visit 10 (Day 50 ± 2 and Day 57 ± 2) of Part A and were determined to be eligible at Visit 10 (Day 57 ± 2) of Part A were allowed to participate in Part B. Those who were not eligible for Part B at Visit 10 (Day 57 ± 2) of Part A were also allowed to enter Part B if they were found eligible by Visit 10 + 7 days of Part A. Part B consisted of a treatment period, a follow‐up period, and a durability observation period. One treatment cycle of zuranolone was defined as a treatment period (2 weeks) and a follow‐up period (6 weeks), with the maximum number of treatment cycles capped at six. No study drug intervention was allowed during the 6‐week follow‐up period. If a participant met the criteria for starting treatment in Part B at the final visit of Part A (Visit 10 [Day 57 ± 2]) or at the end of the follow‐up period (Visit 6 [Day 57]) in each treatment cycle of Part B, they entered the treatment period (2 weeks), followed by a 6‐week follow‐up period (one treatment cycle).

If a participant did not meet the criteria for starting the treatment period of Part B on the reference date, defined as the final visit of Part A (Visit 10 [Day 57]) or at the end of the follow‐up period of the last treatment cycle of Part B (Visit 6 [Day 57]), they entered the durability observation period of 8 weeks. No study intervention was administered during the durability observation period, in which participants were evaluated remotely for the first 7 weeks and at the hospital visit on Week 8 ± 5 days, including a treatment decision visit. This durability observation period was repeated until the re‐treatment criteria were met. The durability observation period comprised remote assessments (Weeks 1, 3, 5, and 7 [±1 day]), remote examination/telephonic consultation, and so forth (Weeks 2 and 6 [±2 days]), remote examination/telemedicine (or visit) (Week 4 [±5 days]), a Week‐8 visit (±5 days]), and a treatment decision visit. In addition, there was an end‐of‐study visit (±7 days) and a termination visit during the durability observation period (Table S1).

**Figure 1:**
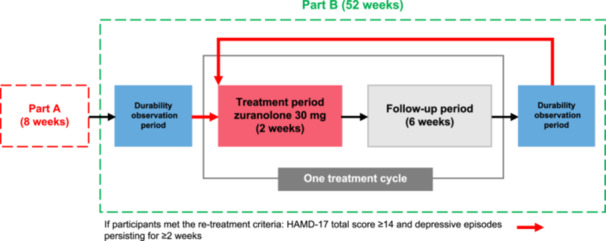
Study design. HAMD‐17, 17‐item Hamilton Depression Rating Scale.

Re‐treatment details and treatment cycles: If the criteria for starting the treatment period of Part B were met, participants returned to the clinic for in‐person assessment within 1 week (assessment of the HAMD‐17 total score by remote examination was not permitted for eligibility) and, if confirmed, entered the treatment period. Additionally, after completion of Part A or any cycle of Part B, if the Patient Health Questionnaire‐9 (PHQ‐9) total score evaluated by the participant using a tablet computer was ≥10 points, or if the physician suspected that treatment might be required, the participant returned to the study site within 1 week for a treatment decision visit and was assessed using the HAMD‐17 scale for the Part B re‐treatment eligibility criteria. Thereafter, the participant had to initiate the 14‐day treatment within 1 week of the site visit, followed by a 6‐week follow‐up to complete one cycle of 8 weeks. Participants had to meet the criteria for re‐treatment at each treatment decision visit after the 8‐week cycle (±5 days), irrespective of the delay in meeting the re‐treatment criteria during the durability observation period after each cycle. The first day of the study intervention in each treatment cycle was defined as Day 1, and a maximum of six treatment cycles were permitted. Participants received the study drug once daily from Day 1 to Day 14, and no dose modification was permitted (Figure 1).

### Participants

Patients who participated in Part A of the study and completed the treatment and follow‐up periods were included. Part A of the study was designed to evaluate the safety and efficacy of zuranolone in patients with MDD. In Part A, eligible participants were Japanese adults (aged 18–75 years) currently diagnosed with MDD based on DSM‐5 criteria, in whom the current MDD episode was ongoing for at least 8 weeks and for ≤12 months. They were also required to have a HAMD‐17 total score of ≥22 and a PHQ‐9 score of ≥15. Participants were excluded if they had evidence of serious comorbid medical conditions, had treatment‐resistant depression, used antidepressants, were treated with device‐based therapies, had a comorbidity or medical history of any other disorder classified under DSM‐5, had suicidal ideation, and were allergic to zuranolone, allopregnanolone, or related substances.

In Part B, female participants were eligible for participation if they were not pregnant or breastfeeding, were not of childbearing potential, or agreed to use highly effective contraceptive methods during the study. Male participants had to refrain from sperm donation and had to agree to use a male condom during the study. Participants received lectures on the use of highly effective contraceptive methods to protect their female partners. All eligible participants had to finish their dinner or a light meal before bedtime, sleep at night, and maintain their regular routine until study completion. Participants judged as ineligible by the investigator due to unresolved adverse events that occurred in Part A were excluded from Part B of the study. Participants with serious hepatic disorder, renal disorder, cardiac disease, pulmonary disease, hematologic disease, metabolic disease, epilepsy (including history of epilepsy), sleep apnea syndrome, interstitial pneumonia, severe bronchial asthma, alveolar hypoventilation syndrome, chronic respiratory failure, pulmonary hypertension, or any other chronic respiratory diseases and ineligible for the study in the opinion of a physician were excluded. Participants who had undergone gastric bypass surgery, gastric sleeve surgery, lap band surgery, or any related procedures that interfered with gastrointestinal transit; had a QTcF > 450 ms; showed an inability to transition to Part B due to abnormalities in the 12‐lead electrocardiogram and medical examinations or had abnormal laboratory test results performed in Part A (physician's discretion); or exhibited suicidal risk (patients who answered “Yes” in Part A to Suicidal Ideation Questions 4 or 5 or to any of the Suicidal Behavior Questions [excluding questions about self‐injurious behavior without suicidal intent] of the Columbia‐Suicide Severity Rating Scale [C‐SSRS]) were also excluded from the study.

### Intervention

During the treatment period, participants received one zuranolone 30‐mg capsule once daily for 14 days to be taken within one hour after dinner. Details of the criteria for study drug continuation, prohibited prior/concomitant therapy, restrictions on prior/concomitant therapy, general restrictions, and criteria for study drug discontinuation are summarized in Table S2.

### Outcomes

Outcomes included the following: (1) treatment‐emergent adverse events (TEAEs) were assessed at all timepoints (visits) during the treatment and follow‐up period, and at the follow‐up visit at the time of treatment discontinuation. In addition, drug dependence or withdrawal symptoms were assessed by the Dependency Assessment Committee; (2) efficacy assessed by number of treatment cycles (maximum six); (3) mean change from baseline in the HAMD‐17 total score among all participants as well as the HAMD‐17 total score (among responders/non‐responders on Day 15 in Part A) were evaluated on Days 8 and 15 of the treatment period and on Days 22, 36, and 57 of the follow‐up period in each treatment cycle; (4) response rate, defined as ≥50% reduction from baseline in the HAMD‐17 total score; and remission rate, defined as HAMD‐17 total score ≤ 7, in each treatment cycle; (5) days to first HAMD‐17 remission; (6) number of treatment cycles among responders on Day 15 of Part A with 1‐year follow‐up data; (7) changes in Clinical Global Impression–Improvement (CGI‐I) scores and change from baseline in Insomnia Severity Index (ISI) total scores in each treatment cycle on Days 8, 15, 22, 36, and 57, and the last observation; and (8) plasma zuranolone concentration (PK) in Cycle 1 and Cycle 2 of Part B.

### Statistical analysis

The full analysis set comprised participants who took at least one dose of the study intervention, including that in Part A, and had a measured HAMD‐17 for at least one time point in Part B. All statistical analyses were performed without imputing the missing values. The total HAMD‐17 score and change from baseline were summarized as mean and standard deviation (SD) at each assessment time point. The HAMD‐17 response and remission rates were calculated as the number and percentage of participants with response and remission, respectively, at each assessment time point. The time to first HAMD‐17 remission was estimated using the Kaplan–Meier method. The CGI‐I rate was calculated as the number and percentage of participants who achieved an improvement in the CGI‐I score at each assessment time point. The absolute and change from baseline ISI scores were summarized as the mean ± SD at each assessment time point. Plasma zuranolone concentrations were determined from the PK samples collected from each patient. All analyses were performed using SAS version 9.4 or higher (SAS Institute, Inc., Cary, NC, USA).

## RESULTS

### Patient disposition

Of the 183 participants in the zuranolone group and 194 participants in the placebo group who completed Part A, 146 and 162 participants provided consent for Part B, respectively. A total of 128 and 143 participants were categorized into zuranolone/zuranolone and placebo/zuranolone groups, respectively, and evaluated in treatment Cycle 1 of Part B (full analysis set/full safety analysis set). Among those 271 participants who were treated in Cycle 1, 213, 170, 147, 126, and 99 participants were re‐treated and constituted the analysis set for safety in Cycles 2, 3, 4, 5, and 6, respectively. The proportion of participants in the full analysis set who completed the 1‐year follow‐up after the first dose of zuranolone in Part B was 67.9% (184/271). Details of patient disposition are presented in Figure S1.

### Demographics and baseline characteristics

The mean (SD) age of participants in treatment Cycle 1 was 39.9 (11.9) years; all were Asian, and 50.2% were males. The mean (SD) baseline HAMD‐17 total score in treatment Cycle 1 was 19.3 (3.7) and similar between the zuranolone/zuranolone (19.2 [3.7]) and placebo/zuranolone (19.4 [3.7]) groups (Tables 1 and S3).

**Table 1:** Demographics and baseline characteristics of participants in Cycle 1 in Part B.

Characteristics	Total *N* = 271 *n* (%)
Sex	
Male	136 (50.2)
Female	135 (49.8)
Age, years	
Mean (SD)	39.9 (11.9)
Median (range)	39.0 (18–68)
Age group, years	
≥18 to <25	29 (10.7)
≥25 to <45	140 (51.7)
≥45 to <65	99 (36.5)
≥65	3 (1.1)
BMI, kg/m^2^	
Mean (SD)	23.3 (4.6)
Race	
Asian	271 (100.0)
HAMD‐17 total score	
Mean (SD)	19.3 (3.7)
Median (range)	19.0 (14–31)
≤24	245 (90.4)
≥25	26 (9.6)
PHQ‐9 total score	
Mean (SD)	15.4 (4.8)
Median (range)	16.0 (1–27)
DSM‐5 classification	
Single episode	104 (38.4)
Recurrent	167 (61.6)
Episode recurrences	
1st time	104 (38.4)
2nd time	96 (35.4)
3rd to 7th time	70 (25.8)
≥8 times	0
Unknown	1 (0.4)
Duration of current episode at randomization, months	
Mean (SD)	5.89 (2.86)
Median (range)	5.19 (2.2–12.4)
2–4	100 (36.9)
4–6	61 (22.5)
6–8	45 (16.6)
8–10	27 (10.0)
10–12	35 (12.9)
≥12	3 (1.1)
Prior drug therapies for depressive episodes	
Yes	150 (55.4)
No	121 (44.6)
Previous disease	26 (9.6)
Concurrent disease	194 (71.6)
Employment status	
Full‐time (≥35 h per week)	99 (36.5)
Part‐time (<35 h per week)	37 (13.7)
Unemployed	60 (22.1)
Retired	18 (6.6)
Other	57 (21.0)

*Note*: The analysis was performed according to the planned study intervention in Part A. The baseline values were the data collected at baseline in Part A, except for weight, BMI, HAMD‐17, and PHQ‐9. The full analysis set included all participants who received at least one dose of the study intervention, including that in Part A, and had HAMD‐17 measured at least one time point in Part B.

Abbreviations: BMI, body mass index; DSM‐5, Diagnostic and Statistical Manual of Mental Disorders, 5th edition; HAMD‐17, 17‐item Hamilton Depression Rating Scale; PHQ‐9, Patient Health Questionnaire‐9; SD, standard deviation.

### Safety

TEAEs occurring in ≥5% of the participants at any time during the treatment period were somnolence (33/271 [12.2%]) and dizziness (23/271 [8.5%]). During the follow‐up period, the only TEAE observed in ≥5% of the participants at any time was nasopharyngitis. No deaths were reported in this study. Although the number of study participants included in the safety analysis set for each number of treatment cycles in Part B was different, the incidence of TEAEs during the treatment period did not show an increase with the number of treatment cycles. According to the assessment of the Drug Dependence Assessment Committee, none of the participants met the drug dependence criteria. The Committee reviewed these data along with participants' clinical conditions; Drug Effect Questionnaire‐5 (DEQ‐5) data; efficacy data, including HAMD‐17 and PHQ‐9 total scores; and other safety data, concluding that no cases were considered drug dependence. In addition, no TEAEs suggestive of drug dependence or withdrawal symptoms were identified, and no TEAEs of suicidal ideation or behavior were reported (Table 2). The incidence and severity of each event were similar across all treatment cycles in Part B (Table S4).

**Table 2:** TEAEs by total treatment cycles in Part B (safety set).

	Total treatment cycles in Part B					
	1	2	3	4	5	6
	(*n* = 271)	(*n* = 213)	(*n* = 170)	(*n* = 147)	(*n* = 126)	(*n* = 99)
*Treatment period*						
Participants with any TEAE, *n* (%)	104 (38.4)	59 (27.7)	40 (23.5)	35 (23.8)	31 (24.6)	15 (15.2)
Severe	1 (0.4)	2 (0.9)	0 (0)	0 (0)	0 (0)	0 (0)
Moderate	20 (7.4)	6 (2.8)	6 (3.5)	8 (5.4)	2 (1.6)	2 (2.0)
Mild	83 (30.6)	51 (23.9)	34 (20)	27 (18.4)	29 (23.0)	13 (13.1)
TEAEs leading to study drug discontinuation (events)	10	1	0	0	0	0
TEAEs in ≥5% of subjects at any time, *n* (%)						
− Somnolence	33 (12.2)	12 (5.6)	4 (2.4)	4 (2.7)	3 (2.4)	2 (2.0)
− Dizziness	23 (8.5)	7 (3.3)	6 (3.5)	5 (3.4)	6 (4.8)	3 (3.0)
*Follow‐up period*						
Participants with any TEAE, *n* (%)	66 (24.4)	43 (20.2)	34 (20)	20 (13.6)	16 (12.7)	24 (24.2)
Severe	0 (0)	1 (0.5)	0 (0)	0 (0)	1 (0.8)	0 (0)
Moderate	17 (6.3)	11 (5.2)	9 (5.3)	6 (4.1)	6 (4.8)	8 (8.1)
Mild	49 (18.1)	31 (14.6)	25 (14.7)	14 (9.5)	9 (7.1)	16 (16.2)
TEAEs in ≥5% of subjects at any time, *n* (%)						
Nasopharyngitis	9 (3.3)	6 (2.8)	7 (4.1)	0 (0)	2 (1.6)	7 (7.1)

Abbreviation: TEAE, treatment‐emergent adverse event.

The majority of TEAEs were mild or moderate in severity. In the zuranolone/zuranolone group, one severe event of an altered state of consciousness occurred in one patient in Cycle 1, which was deemed related to the study drug. Three participants in the placebo/zuranolone group experienced adverse events that were deemed severe, including spontaneous pneumothorax and mechanical ileus in one participant, and abnormal liver function test results occurred in a second participant in Cycle 2, and malignant pleural effusion occurred in a third participant in Cycle 5. Of these, the TEAE of abnormal liver function test results was deemed to be related to the study drug.

Overall, in Part B, there were 10 events occurring in six participants that were considered to be serious TEAEs, four in the zuranolone/zuranolone group (three participants), and six in the placebo/zuranolone group (three participants). There were four events of serious TEAEs (altered state of consciousness, large intestine polyp and cerebral infarction, and positional vertigo) occurring in three participants in the zuranolone/zuranolone group. Altered state of consciousness occurred in a 22‐year‐old female who intentionally overdosed on 11 capsules of zuranolone 30 mg on Day 4 of the treatment period in Cycle 1, after an argument with her mother. The participant was hospitalized on the evening of the event and treated with intravenous fluids only. The overdose was not considered a suicide attempt based on C‐SSRS data, and the principal investigator confirmed that there was no suicide attempt following the overdose. On examination, no remarkable abnormal findings were reported. The event was assessed to be temporally related to the study drug, and the participant was discharged from the hospital after 2 days following full recovery. A large intestinal polyp and cerebral infarction occurred in a female participant. The large intestinal polyp resolved after polypectomy, and the patient continued to participate in the study. This event was considered unrelated to the study intervention. Cerebral infarction required hospitalization and treatment for 10 days, and the event was considered resolving at discharge. This participant was withdrawn from the study. The investigator assessed this event to be unrelated to the study medication. Positional vertigo occurred in a female participant and resolved after hospitalization for 4 days in a participant with a history of sudden hearing loss who experienced vertigo and vomiting approximately 3 weeks after the completion of the study intervention. This event was considered unrelated to the study intervention.

Six events of serious TEAEs (COVID‐19 and abnormal liver function test, mechanical ileus and spontaneous pneumothorax, and malignant pleural effusion and invasive ductal breast carcinoma) occurred in three participants in the placebo/zuranolone group. A male participant was diagnosed with COVID‐19 2 weeks after the initiation of the study intervention and was hospitalized for 6 days. The event resolved 6 days after hospitalization. The TEAE of abnormal liver function test results occurred on Day 8 of the treatment period in Cycle 2 in a male patient aged 53 years. However, this was due to an alternative cause; the participant had consumed a large amount of alcohol the day before the investigation (Day 7 of treatment), and the result was assessed to be temporally related to the study intervention. Mechanical ileus and spontaneous pneumothorax after completing the 14‐day zuranolone treatment in Cycle 2 were considered to have occurred accidentally due to participant‐related factors including Hirschsprung's disease and abdominal operation history. The participant was withdrawn from the study and recovered from the TEAEs. Malignant pleural effusion and invasive ductal breast carcinoma were diagnosed in Cycle 5, and the participant was admitted. This participant was withdrawn from the study, and the investigator considered malignant pleural effusion to be resolving and left invasive ductal breast carcinoma as not recovered.

The overall incidence of TEAE occurrence was comparable between males (40/98 [40.8%]) and females (46/101 [45.5%]) (Table S5).

Overall, eight participants had TEAEs leading to discontinuation of the study intervention. Four TEAEs leading to the discontinuation of study intervention occurred in two participants (1.4%) in the zuranolone/zuranolone group (one event each of headache, dizziness, malaise, and altered state of consciousness) and seven TEAEs leading to discontinuation of study intervention occurred in six participants (3.8%) in the placebo/zuranolone group (one event each of abnormal liver function test, feeling drunk, akathisia, palpitation, nausea, dizziness, and somnolence). All cases were of mild or moderate severity, except, as mentioned above, the events of altered state of consciousness and abnormal liver function test results, which were severe. All TEAEs leading to study discontinuation were resolved or resolving and were considered to be related to the study intervention.

### Efficacy

**Figure 2:**
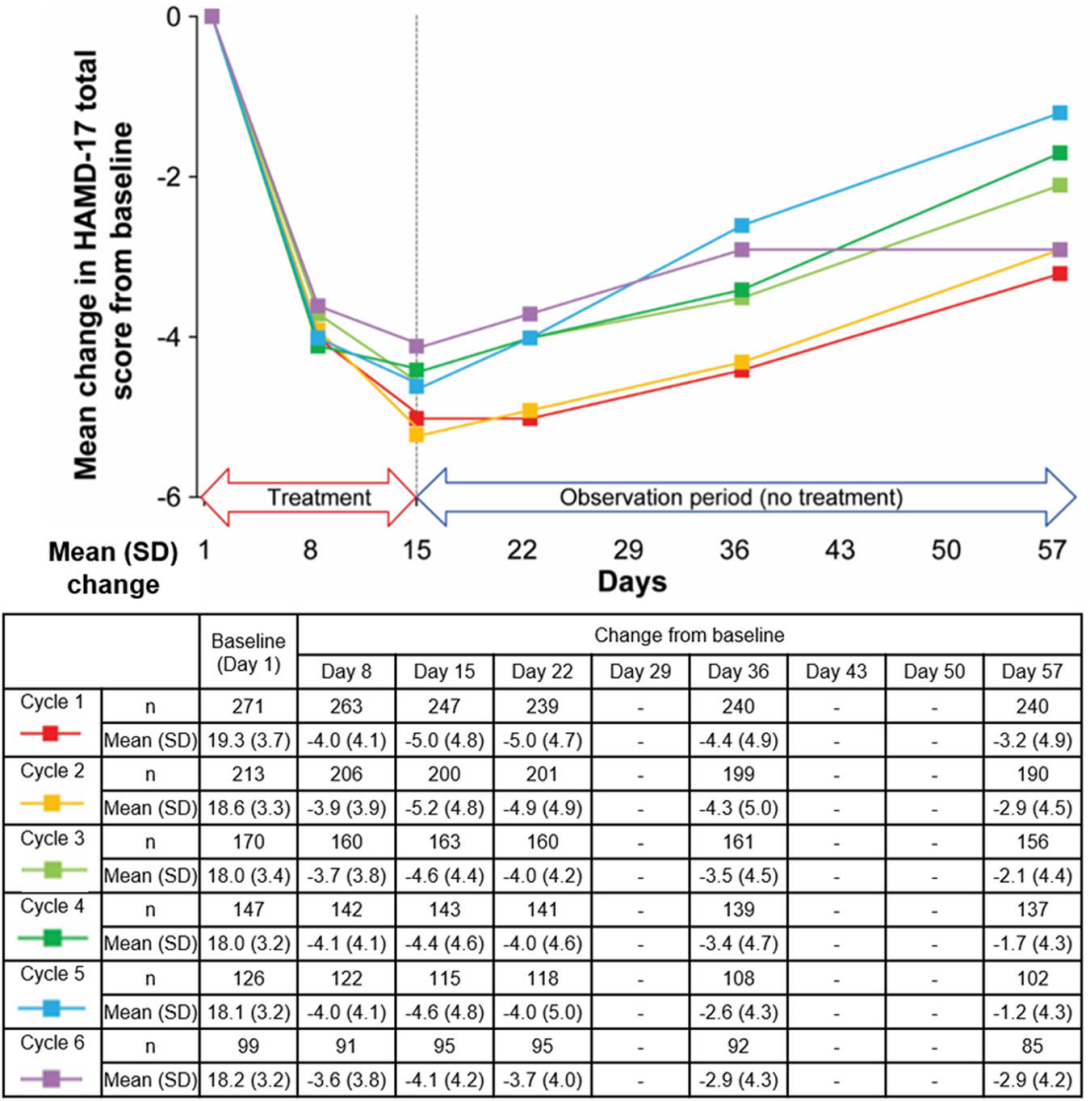
Mean change in HAMD‐17 total score from baseline in Part B of the study (full analysis set). HAMD‐17, 17‐item Hamilton Depression Rating Scale; SD, standard deviation.

The mean (SD) HAMD‐17 total score decreased compared with baseline at Day 8 (range: −3.6 [3.8] to −4.1 [4.1]) and Day 15 (range: −4.1 [4.2] to −5.2 [4.8]) of the treatment period in all six treatment cycles. However, no further decrease was observed during the observation period (Days 22–57) of any of these cycles (range: −1.2 [4.3] to −3.2 [4.9] on Day 57) (Figure 2 and Table S6). The mean (SD) change from baseline in HAMD‐17 total score, categorized into the zuranolone/zuranolone and placebo/zuranolone groups, is presented in Figure S2a,b.

Among participants who responded to treatment on Day 15 in the zuranolone group in Part A, the magnitude of response was consistently greater compared with that observed for the total zuranolone/zuranolone group of Part B, and remained similar across all six treatment cycles of Part B. Reduction (change from baseline) in mean (SD) HAMD‐17 total scores were observed on Day 8 (range: −16.0 [NA] to −8.4 [4.3]) and Day 15 (range: −16.0 [NA] to −10.9 [2.7]) during the treatment period. These reductions gradually decreased during the observation period (Days 22–57) in all six treatment cycles (range: −11.0 [NA] to −2.7 [8.5] on Day 57) (Figure S2c). Among non‐responders to treatment on Day 15 in the zuranolone group in Part A, although reductions (change from baseline) in mean (SD) HAMD‐17 total scores were observed on Day 8 (range: −3.6 [3.7] to −2.1 [2.6]) and Day 15 (range: −4.6 [4.5] to −2.8 [2.7]) during the treatment period, the reductions in scores were smaller than those of responders to the initial zuranolone treatment (Figure S2e).

Among responders to the initial zuranolone treatment on Day 15 in Cycle 1 of Part B, the response was consistently greater compared with that in all participants in the placebo/zuranolone group of Part B and was similar across all six treatment cycles of Part B. Reductions (change from baseline) in the mean (SD) HAMD‐17 total scores were observed on Day 8 (range: −5.2 [4.3] to −8.9 [4.5]) and Day 15 (range: −7.2 [4.0] to −12.3 [3.0]) of the treatment period. These reductions gradually decreased during the observation period (Days 22–57) in all six treatment cycles (range: −2.2 [4.3] to −4.6 [5.5] on Day 57) (Figure S2d). Among non‐responders to the initial zuranolone treatment on Day 15 in Cycle 1 of Part B, although reductions (change from baseline) in mean (SD) HAMD‐17 total scores were observed on Day 8 (range: −3.9 [3.7] to −2.6 [3.1]) and Day 15 (range: −4.6 [3.9] to −3.2 [3.5]) during the treatment period, the reductions in scores were smaller than those of responders to the initial zuranolone treatment (Figure S2f).

A total of 271, 213, 170, 147, 126, and 99 participants were evaluated in Cycles 1–6, respectively, and there were 58, 43, 23, 21, 27, and 99 participants who had received 1, 2, 3, 4, 5, and 6 total treatment cycles, respectively, in Part B. Mean percentage change in HAMD‐17 total score was evaluated by total number of treatment cycles. Comparing HAMD‐17 at each treatment cycle among participants who required the same number of treatment cycles in Part B, the decrease in HAMD‐17 total score was similar in each treatment cycle (Figure S3a–f).

**Figure 3:**
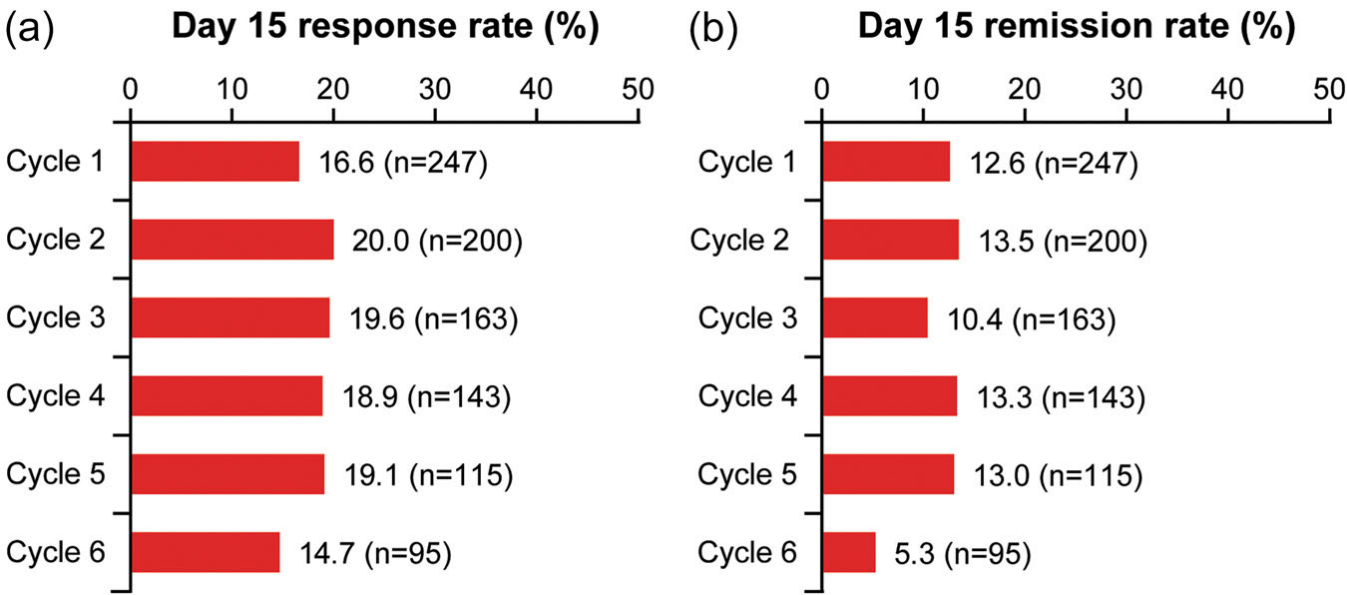
HAMD‐17 response rates and remission rates on Day 15 in each treatment cycle of Part B. HAMD‐17, 17‐item Hamilton Depression Rating Scale.

The response rate on Day 15 was similar across all treatment cycles: Cycle 1 (16.6%), Cycle 2 (20.0%), Cycle 3 (19.6%), Cycle 4 (18.9%), Cycle 5 (19.1%), and Cycle 6 (14.7%) (Figure 3a). A similar remission rate was observed for each repeated treatment cycle: Cycle 1 (12.6%), Cycle 2 (13.5%), Cycle 3 (10.4%), Cycle 4 (13.3%), Cycle 5 (13.0%), and Cycle 6 (5.3%) (Figure 3b). The response rate and remission rate were consistent when categorized into zuranolone/zuranolone and placebo/zuranolone groups (Figure S4).

There were 184 participants who completed the 1‐year follow‐up from their first treatment with zuranolone. Of the 184 participants, approximately half (91/184, 49.5%) received the maximum number of treatment cycles (six cycles in Part B).

At the 1‐year follow‐up period, there were 41 participants with a response on Day 15 of the first treatment with zuranolone. Among these 41 participants who responded to initial zuranolone treatment, 11 (26.8%) received six treatments in Part B, a lower percentage than all participants with 1‐year follow‐up from their first treatment with zuranolone (Table S7).

Figure S5 shows the number of participants who experienced initial remission over time. Approximately 20% of participants achieved initial remission (HAMD‐17 total score ≤7) at the end of the 6‐week follow‐up after the first treatment initiation in Part A. As a result of repeated treatment, approximately 40% of patients achieved initial remission within half a year and approximately 60% achieved initial remission at 1 year. The number of participants experiencing first remission increased with each repeated treatment cycle (Figure S5).

CGI‐I scores improved during the 14‐day treatment period (Days 1–15) in all treatment cycles, and the scores on Day 15 tended to decrease thereafter during the follow‐up period (Days 22–57) except in Cycle 2, where the scores improved until Day 22. The treatment response rate assessed based on improvement in the CGI‐I score on Day 15 in all six treatment cycles ranged from 27.1% to 33.9% (Table S8).

Improvement in insomnia was observed as a decrease in ISI scores from baseline during the 14‐day treatment period (Days 1–15) in all six treatment cycles. Thereafter, during the follow‐up period (Days 22–57) in all treatment cycles, the ISI scores tended to increase but did not exceed baseline levels.

The mean baseline ISI scores for each treatment cycle were similar and ranged from 15.9 to 16.7. The mean change from baseline in ISI scores for Day 15 in each treatment cycle ranged from −3.5 to −4.1 (Table S9).

### Pharmacokinetics

In Part B, 509 plasma samples were collected from 271 participants in treatment Cycle 1, and 406 plasma samples were collected from 212 participants in treatment Cycle 2. Zuranolone was absorbed orally, and there were no clinically meaningful differences in plasma concentrations between Day 8 and Day 15 of exposure to zuranolone in treatment Cycles 1 and 2 (Figure S6).

## DISCUSSION

Part B of this Phase 3 study was conducted to evaluate the safety and tolerability of repeated 14‐day courses of zuranolone followed by a 6‐week follow‐up period in each (8‐week) treatment cycle for a maximum of six treatment cycles over 1 year in Japanese patients with MDD. Most TEAEs that occurred in this study were mild or moderate in severity, and no new safety signals were observed. The types of TEAEs and their incidence rates in the initial zuranolone treatment cycle and repeated treatment cycles were similar. TEAEs occurring in ≥5% of participants during the treatment period were somnolence and dizziness, which were also observed in ≥5% of participants in Part A (the prior double‐blind trial).^21^ During the 6‐week follow‐up periods, the only TEAE observed in ≥5% of participants was nasopharyngitis. The incidence and severity of each event were similar between Part A and all treatment cycles in Part B. However, small differences were noted in the incidence of TEAEs across individual treatment cycles. Ten participants discontinued treatment due to TEAEs during Cycle 1, one during Cycle 2, and none during subsequent cycles. The decline in the reported TEAEs across cycles is likely attributable to selective attrition, as participants who experienced intolerable adverse events early on were withdrawn from the study.

Adverse events often associated with antidepressant use—including nausea, insomnia, somnolence, fatigue, weight gain, sexual dysfunction, and suicidality—were not frequently observed in the current study.^22^, ^23^ Specifically, the frequency of nausea and vomiting was low, and there were no reports of weight gain, sexual dysfunction, or suicidality with zuranolone use in this study. There were no deaths reported in this study.

In Part B, there were four serious TEAEs (altered state of consciousness due to zuranolone overdose, large intestine polyp and cerebral infarction, and positional vertigo) in three participants in the zuranolone/zuranolone group and six serious TEAEs (COVID‐19 and abnormal liver function test, mechanical ileus and spontaneous pneumothorax, malignant pleural effusion, and invasive ductal breast carcinoma) in three participants in the placebo/zuranolone group. Of these, an altered state of consciousness and abnormal liver function test results were considered by the investigator to be related to the study drug and resolved in 2 and 17 days, respectively.

Although a warning for suicidal thoughts and behavior is included in the package insert in the United States,^9^ based on the C‐SSRS, no suicidal thoughts and behaviors were identified in this study, consistent with previous reports in domestic Japanese studies.^21^ No TEAEs suggestive of drug dependence or withdrawal symptoms were identified. In this study, withdrawal symptoms might not have been detected because a specific evaluation scale was not employed, or the participants' moderate to severe depression could have obscured these symptoms.

In the United States, zuranolone has the same controlled substance scheduling (Schedule IV) as benzodiazepines. A previous study showed that supratherapeutic doses of zuranolone have mild abuse liability properties similar to those when using alprazolam, a Schedule IV benzodiazepine.^24^ However, no cases of apparent physical dependence were identified in this study.

Zuranolone acts on the neurosteroid pathway. Allopregnanolone levels in females are similar to those in men during the follicular phase, but rise significantly during the luteal (premenstrual) phase, suggesting that hormonal status may influence adverse events or treatment response.^25^, ^26^ No clinically meaningful sex‐based differences were observed in the HAMD‐17 responses during Part A of this study,^21^ and evaluation of premenopausal vs. postmenopausal women was hampered by the reduced sample size and wider confidence intervals within each group. Likewise, in Part B of the study, there were no clear differences in HAMD‐17 response between menstruating females and menopausal females, nor in TEAE incidence. Additionally, the study did not prohibit the use of hormone‐based contraceptives in premenopausal women, which may have further affected the ability to evaluate the potential impact of hormonal fluctuations on zuranolone efficacy or safety in female patients. Additional investigation is therefore warranted to evaluate this question.

Similar results were observed in the safety and the Day 15 response rate in Part B between the groups that received zuranolone treatment or placebo in Part A.^21^ Clinical response, as measured by a 50% reduction in HAMD‐17 total score, has been shown to correlate strongly with clinically meaningful symptom improvement.^27^, ^28^ Based on these results, repeated administration of zuranolone resulted in clear, clinically meaningful improvement in 14.7%–20.0% of participants per treatment cycle. There were no obvious differences in plasma concentrations between Days 8 and 15 compared with those in previous studies.^19^, ^21^ The reduction in HAMD‐17 total score at Day 15 was observed in either group in all treatment cycles in Part B. The treatment response rate assessed based on the CGI‐I scores on Day 15 in all six treatment cycles was similar to the Day 15 response rate in the zuranolone 30‐mg group in Part A (31.3%).^21^ Based on the open‐label design, we were unable to account for the possible contribution of expectation bias on the results. As the majority of discontinuations were not linked to a lack of efficacy (Figure S1), it is unlikely that selective attrition had a significant impact on the reproducibility of drug effect across treatment cycles. In Part B, the number of participants who achieved the first remission increased with an increasing number of repeated treatment cycles. Among responders to the first treatment of zuranolone on Day 15, the response was greater compared with that in all participants in Part B and similar in all six treatment cycles in Part B. When participants were grouped by the same total number of treatment cycles, the reduction in the HAMD‑17 total score was similar across cycles. Participants who did not respond to the initial zuranolone treatment also showed reductions in the HAMD‑17 total scores. Although these reductions were smaller compared to those observed in responders to the initial zuranolone treatment, the cumulative number of participants achieving remission increased with additional treatment cycles.

Among the participants who completed follow‐up for 1 year in Part B, approximately 50% received six treatment cycles. Among the participants who responded to the initial zuranolone treatment in Part A, the total number of treatment cycles was small, with 36.6% of participants receiving ≤3 treatment cycles per year, and many of these participants remained in remission after the last dose. The re‐treatment criterion for this study was set at HAMD‐17 ≥ 14, which is an indicator of moderate to severe depression. In clinical practice, treatment guidelines typically prioritize the recurrence of clinically significant symptoms over a specific HAMD‐17 threshold when making treatment decisions. In this context, our criteria for re‐treatment align with these guidelines, as well as with clinical practice, where patients exhibiting mild to moderate depressive symptoms are considered suitable candidates for treatment.^29^ The proportion of patients who received only one treatment course was 21.4% (58/271). On the other hand, the re‐treatment criterion for the long‐term, open‐label, Phase 3 SHORELINE study of zuranolone in the United States was HAMD‐17 ≥ 20; in that study, the proportion of responders who received only one treatment course was 42.9% in the 30‐mg zuranolone treatment group and 54.8% in the 50‐mg zuranolone treatment group.^20^ The differences in re‐treatment criteria may explain the differences between our study and the SHORELINE study in the proportion of responders who received only one treatment course. Considering that the number of treatments was lower in the SHORELINE trial than in this trial, it is expected that the number of treatments would increase if a lower score were used as the re‐administration criterion. This study design comprised a 2‐week zuranolone treatment period followed by at least 6 weeks of follow‐up period and re‐treatment as needed. Although no other treatments for depression were permitted (such as rescue medication), approximately 60% of the initial responders in this study completed the 1‐year follow‐up. The results of this study suggest that the zuranolone 30‐mg dose, administered using intermittent courses (2 weeks of treatment followed by 6 weeks off‐treatment) as needed, could be a novel therapeutic option for the long‐term treatment of depression.

## LIMITATIONS

As Part B of this Phase 3 study was an open‐label study, there were no comparative data with a placebo to assess the safety and efficacy of repeated treatment cycles. Part B of this study was not designed for comparative safety and efficacy owing to the lack of a control group and absence of randomization; nevertheless, the results of the initial placebo‐controlled portion of the study (Part A) have already been reported.^21^ In the present (Part B) study, participants were re‐treated with zuranolone 30 mg once daily as required (zuranolone/zuranolone group and placebo/zuranolone group) if they met the re‐treatment criteria, respectively: HAMD‐17 total score ≥ 14 and depressive episodes persisting for ≥2 weeks (based on DSM‐5). However, in clinical practice, it is assumed that re‐treatment will be initiated in patients whose initial response reduced depressive symptoms to a level consistent with mild severity or full remission. There are insufficient data to discuss efficacy in such a patient population from this study. This study did not permit the concomitant use of other antidepressants (or rescue medication) and required a 6‐week follow‐up period for zuranolone before re‐treatment. It is assumed that switching to other drugs may be considered within 6 weeks of a follow‐up period; therefore, the design of this study does not reflect a real‐world clinical setting. In this study, no specific assessment scale for withdrawal symptoms was used. Considering the half‐life of zuranolone, any acute discontinuation effect would be expected to emerge within 1 week after treatment discontinuation. Although participants were followed up to 1 year, no late‐onset or protracted withdrawal symptoms were observed. Furthermore, the participants had moderate to severe depression, which may have masked potential withdrawal effects. Additionally, the US ZURZUVAE package insert notes that insomnia, palpitations, decreased appetite, nightmares, nausea, hyperhidrosis, and paranoia have been reported after discontinuation of zuranolone in healthy participants receiving 50 mg of zuranolone for 5–7 days (on Day 7, participants received 50 or 100 mg).^9^ Therefore, further investigation is warranted to better characterize the withdrawal symptoms in Japanese patients, particularly under conditions that may be more consistent with “real‐world” use.

## CONCLUSIONS

In repeated‐treatment cycles of 14‐day zuranolone therapy with a 6‐week follow‐up period for a maximum of six “treatment plus follow‐up cycles” over 1 year, there were no new safety signals. The HAMD‐17 total scores decreased from baseline over 15 days during the treatment period of each repeated treatment cycle. The mean change from baseline in HAMD‐17 total scores at Day 15 ranged from −5.2 to −4.1 in Cycles 1–6, and a reduction in HAMD‐17 total scores was observed in all six treatment cycles.

## AUTHOR CONTRIBUTIONS

**Masaki Kato**: Conception and design, interpretation of results, and review of the manuscript. **Kazuyuki Nakagome**: Conception and design, interpretation of results, and review of the manuscript. **Takamichi Baba**: Conception and design, analysis of data, and review of the manuscript. **Takuhiro Sonoyama**: Conception and design, interpretation of results, and review of the manuscript. **Hiroki Fukuju**: Conception and design, acquisition and analysis of data, and review of the manuscript. **Ryosuke Shimizu**: Conception and design, analysis of data, and review of the manuscript. **Juan Carlos Gomez**: Conception and design, interpretation of results, and review of the manuscript. **Tomoko Motomiya**: Conception and design, interpretation of results, and review of the manuscript. **Takeshi Inoue**: Conception and design, interpretation of results, and review of the manuscript.

## CONFLICT OF INTEREST STATEMENT

T.S., T.B., and R.S. are full‐time employees and own stocks via employee stock ownership society of Shionogi & Co., Ltd. T.M. and H.F. are full‐time employees of Shionogi & Co., Ltd. J.C.G. is a full‐time employee of Shionogi B.V. T.I. has received personal fees from Mochida Pharmaceutical, Takeda Pharmaceutical Co., Ltd., Janssen Pharmaceutical, Novartis Pharma, MSD, Yoshitomiyakuhin, Nipro, Kyowa Pharmaceutical Industry, Viatris, Lundbeck Japan K.K., Boehringer Ingelheim, Ono Pharmaceutical, and Meiji Seika Pharma Co., Ltd.; grants from Daiichi Sankyo and Tsumura; grants and personal fees from Shionogi & Co., Ltd., Otsuka Pharmaceutical Co., Ltd, Sumitomo Pharma Co., Ltd., Mitsubishi Tanabe Pharma, and Eisai; and is a member of the advisory boards of Luye, Shionogi, GlaxoSmithKline, Viatris, and Otsuka Pharmaceutical. M.K. has received grants from AMED, the Japanese Ministry of Health, Labour and Welfare, the Japan Society for the Promotion of Science, SENSHIN Medical Research Foundation, the Japan Research Foundation for Clinical Pharmacology, and the Japanese Society of Clinical Neuropsychopharmacology; consulting fees from Shionogi & Co., Ltd., Sumitomo Pharma Co., Ltd., Otsuka Pharmaceutical Co., Ltd., Lundbeck Japan K.K., and Takeda Pharmaceutical Co., Ltd.; speaker honoraria from Sumitomo Pharma Co., Ltd., Otsuka Pharmaceutical Co., Ltd., Lundbeck Japan K.K., Takeda Pharmaceutical Co., Ltd., Meiji Seika Pharma Co., Ltd., Shionogi & Co., Ltd., Mitsubishi Tanabe Pharma Corporation, Viatris Inc., Eisai Co., Ltd., and Kyowa Pharmaceutical Industry Co., Ltd.; and is in the general management committee for Depression Treatment Guidelines, Japanese Society of Mood Disorders and is the Vice Chairman of the Guideline Development Committee, Japanese Society of Mood Disorders for the past 36 months. K.N. has received grants paid to his institution from Shionogi & Co., Ltd., Sumitomo Pharma Co., Ltd., Otsuka Pharmaceutical Co., Ltd., Janssen Pharmaceutical K.K., Nippon Boehringer Ingelheim Co., Ltd., and AbbVie GK; honoraria from Sumitomo Pharma Co., Ltd. (Speaker, Chair, Advisor), Otsuka Pharmaceutical Co., Ltd. (Speaker, Chair, Advisor), Meiji Seika Pharma Co., Ltd. (Speaker), Janssen Pharmaceutical K.K. (Chair, Speaker‐Panelist), Mitsubishi Tanabe Pharma Corp. (Chair, Advisor), Viatris Pharmaceuticals Japan G.K. (Chair, Consulting), Nippon Boehringer Ingelheim Co., Ltd. (Chair, Advisor, Supervisor), Boehringer Ingelheim International GmbH (Speaker‐Panelist), Kyowa Kirin Co., Ltd. (Speaker), Shionogi & Co., Ltd. (Chair), and Yoshitomiyakuhin Corp. (Chair, Supervisor); and support for transportation to attend meetings from Sumitomo Pharma Co., Ltd., Otsuka Pharmaceutical Co., Ltd., Meiji Seika Pharma Co., Ltd., Janssen Pharmaceutical K.K., Mitsubishi Tanabe Pharma Corp., Nippon Boehringer Ingelheim Co., Ltd., Boehringer Ingelheim International GmbH, Shionogi & Co., Ltd., Yoshitomiyakuhin Corp., and AbbVie GK.

## ETHICS APPROVAL STATEMENT

This study was conducted in compliance with ethical principles based on international guidelines, including the Declaration of Helsinki, Council for International Organizations of Medical Sciences, International Ethical Guidelines, ICH‐GCP guidelines, and other applicable laws and regulations, and was approved by the institutional review board and ethics committee of each study site.^21^

## PATIENT CONSENT STATEMENT

Written informed consent was obtained from each participant after a full explanation of the study procedures and objectives.

## CLINICAL TRIAL REGISTRATION

The study was registered with the Japan Registry for Clinical Trials (jRCT) Clinical Trials Registry before patient enrollment (Study ID: jRCT2031210577).

## Supporting information


**Supporting Information**.

## Data Availability

Shionogi & Co., Ltd. is committed to disclosing the synopses and results of its clinical trials and sharing clinical trial data (raw dataset or study data tabulation model dataset) with researchers upon request. If the research proposal is reviewed and approved by an independent review panel, anonymized data and redacted documents will be provided in a secure research environment. For further details, please refer to the websites of Shionogi & Co., Ltd. (https://www.shionogi.com/global/en/company/policies/shionogi-group-clinical-trial-data-transparency-policy.html) and Vivli (https://vivli.org/).
