# Supplementary material for: Safety and efficacy of zuranolone in Japanese adults with major depressive disorder: An open‐label, repeated‐treatment part of a Phase 3 clinical trial
Source: PCN Rep. 2026 Feb 19;5(1):e70302. doi: 10.1002/pcn5.70302 (PMC12917863; doi:10.1002/pcn5.70302)
Supplement: Supplementary file 1 — Supporting Information. [file PCN5-5-e70302-s001.docx]

**Supplementary Fig. 1** Patient disposition


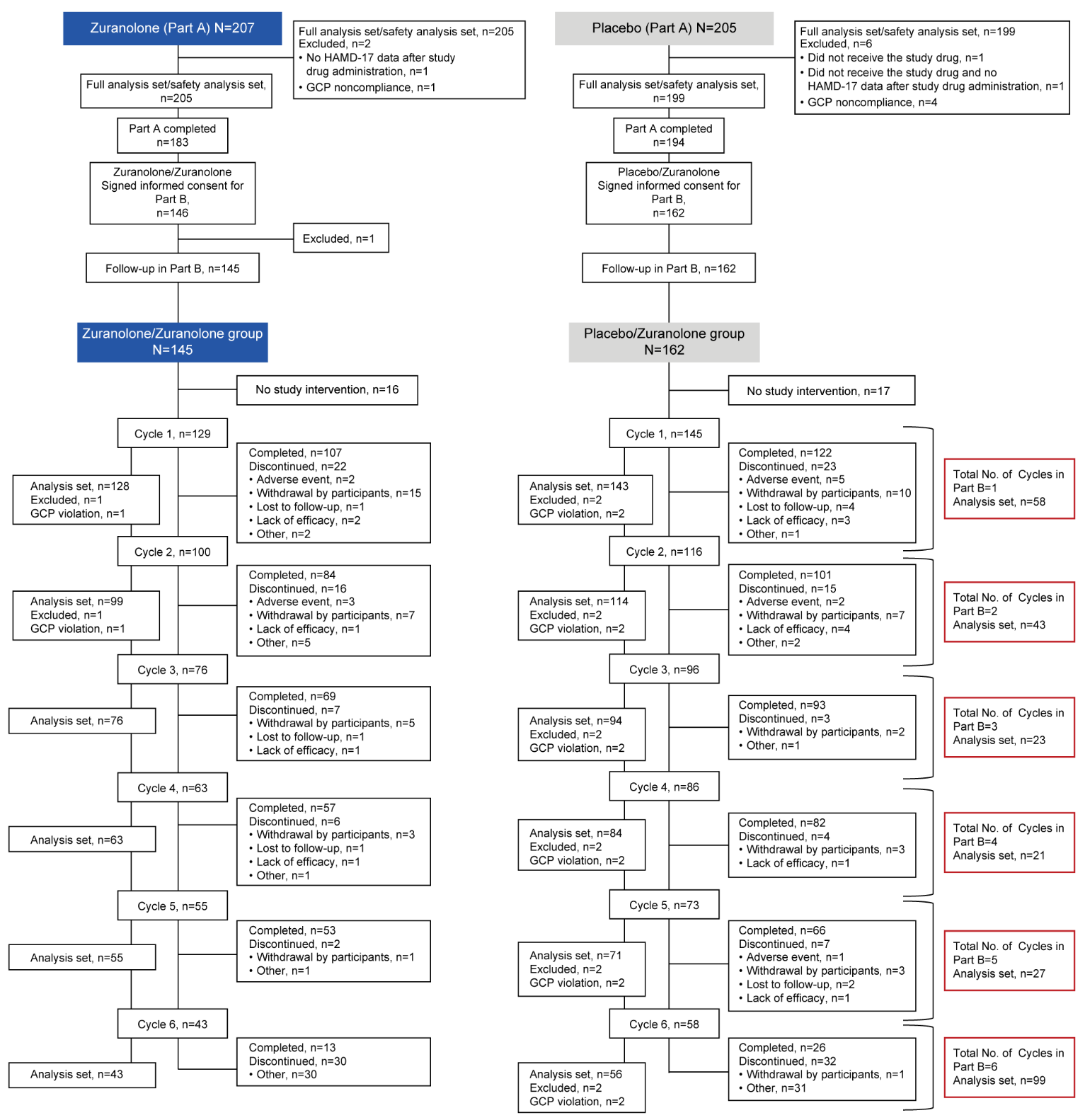


GCP, Good Clinical Practice; HAMD-17, 17-item Hamilton Depression Rating Scale

**Supplementary Fig. 2** Mean change from baseline in HAMD-17 in Part B of the study categorized as (a) Full analysis set of the Zuranolone/Zuranolone group, (b) Full analysis set of the Placebo/Zuranolone group, (c) Responders of the initial zuranolone treatment in Part A, (d) Responders of the initial zuranolone treatment in Part B, (e) Non-responders of the initial zuranolone treatment in Part A, and (f) Non-responders of the initial zuranolone treatment in Part B
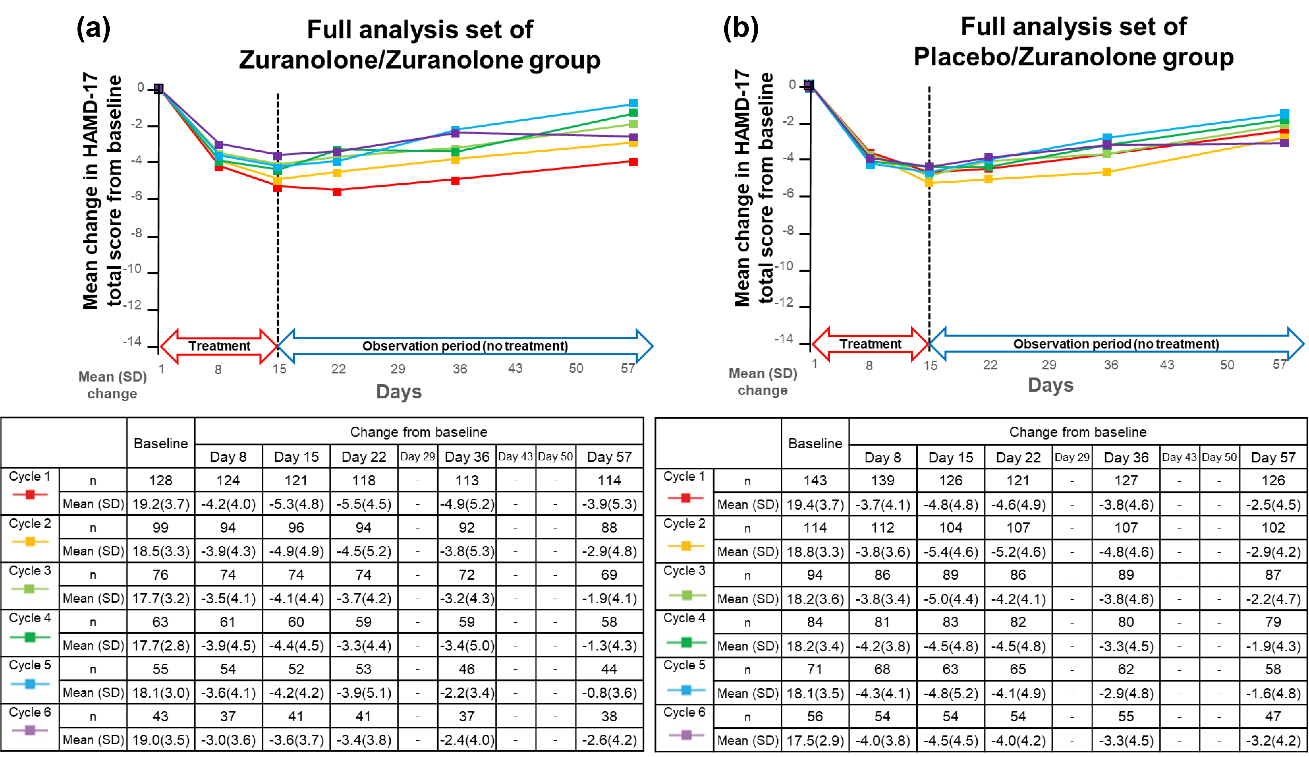


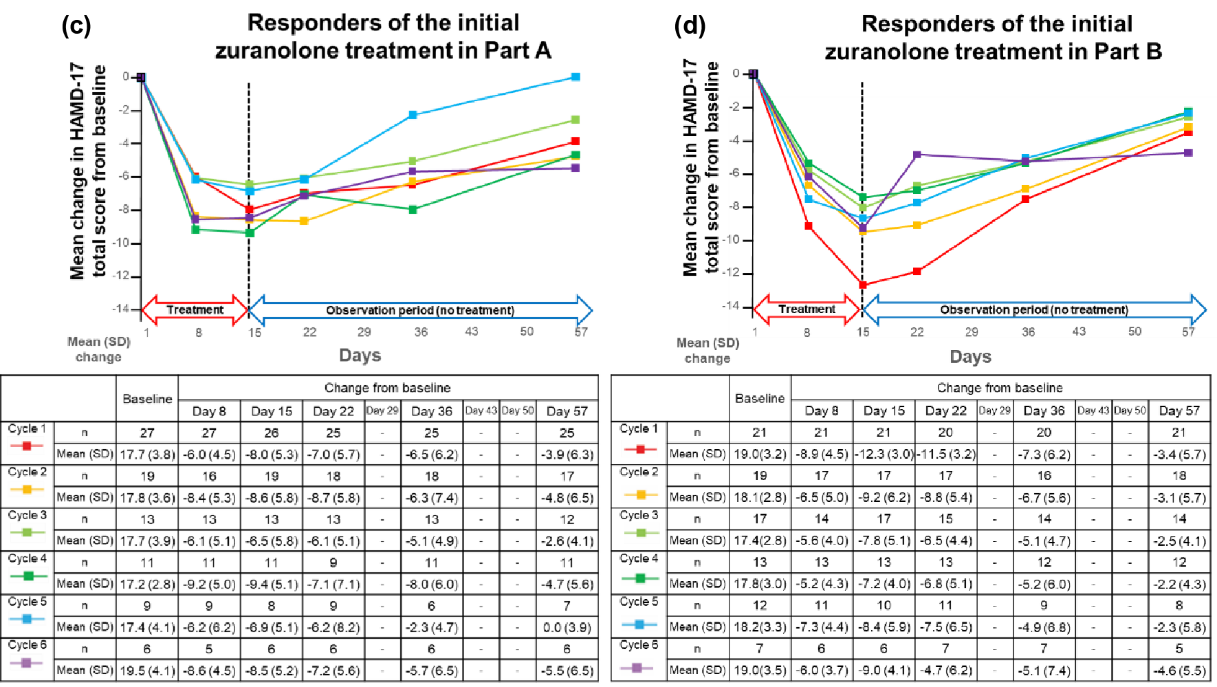

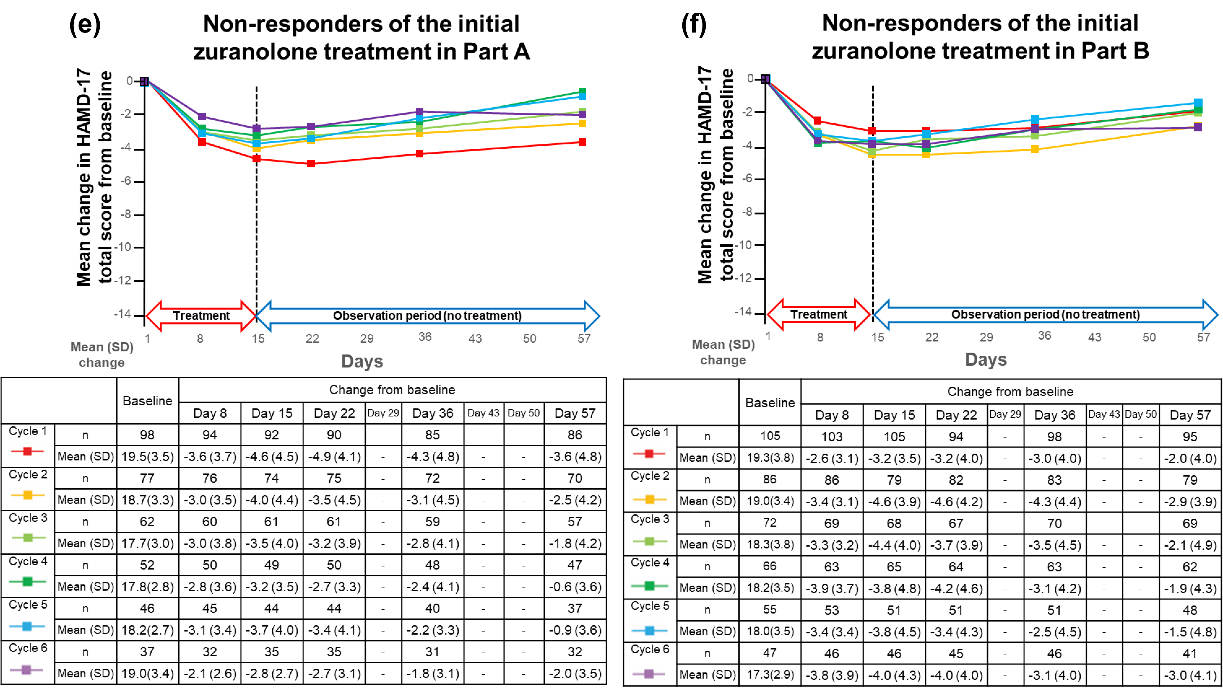
HAMD-17, 17-item Hamilton Rating Scale for Depression

Responders of the initial zuranolone treatment: Participants who had been assigned to the zuranolone group in Part A and showed response on Day 15 with zuranolone in Part A or participants who had been assigned to the placebo group in Part A and showed response on Day 15 with zuranolone in treatment cycle1 of Part B

Non-responders of the initial zuranolone treatment: Participants who had been assigned to the zuranolone group in Part A and did not show response on Day 15 with zuranolone in Part A or participants who had been assigned to the placebo group in Part A and did not show response on Day 15 with zuranolone in treatment cycle1 of Part B

**Supplementary Fig. 3** Mean percentage change in HAMD-17 total score by total treatment cycles in Part B.


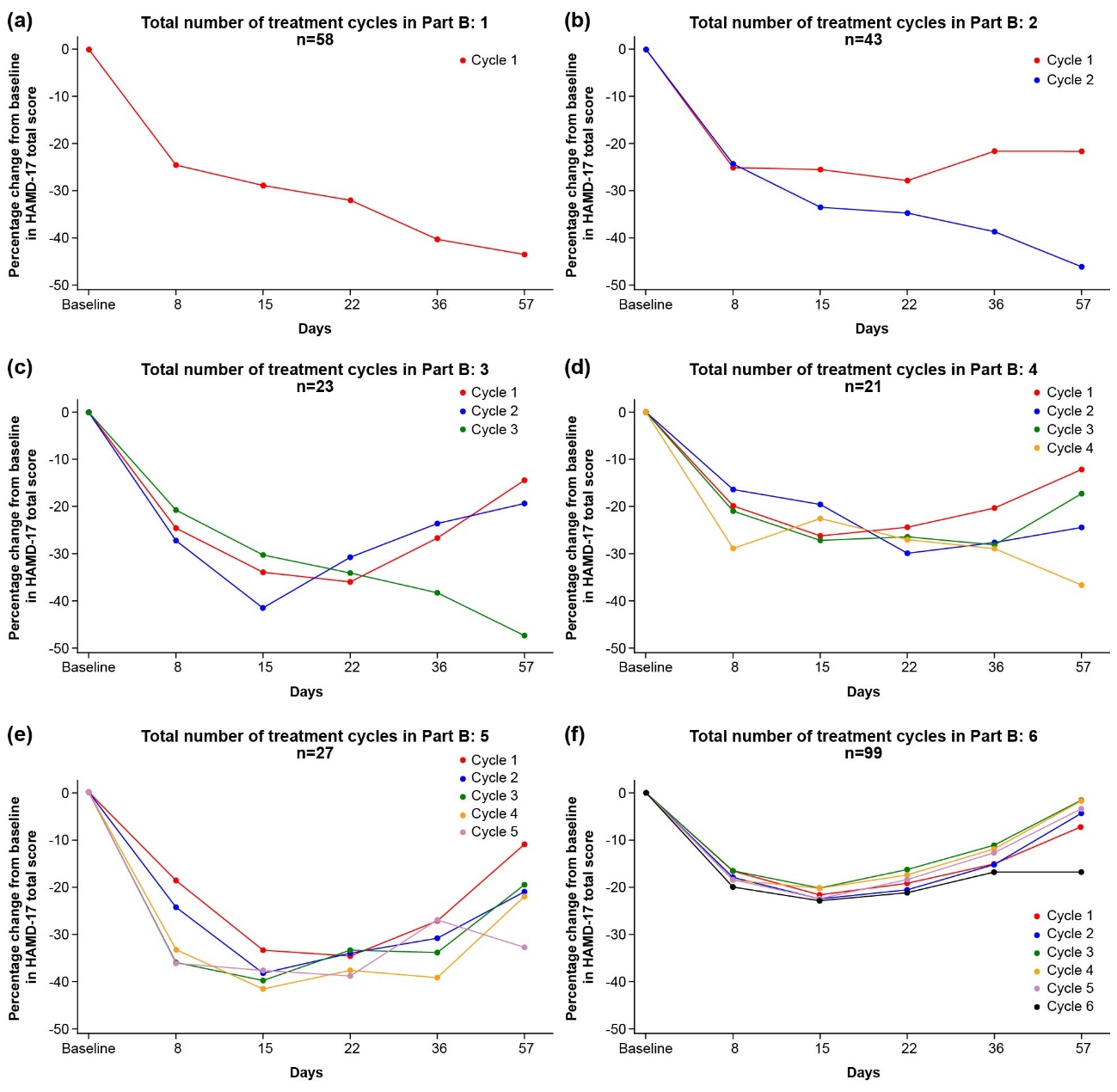


HAMD-17, 17-item Hamilton Rating Scale for Depression

**Supplementary Fig. 4** Plot of HAMD-17 response rates categorized as (a) Zuranolone/Zuranolone group and (b) Placebo/Zuranolone group, and remission rates categorized as (c) Zuranolone/Zuranolone group and (d) Placebo/Zuranolone group on Day 15 in each treatment cycle of Part B.


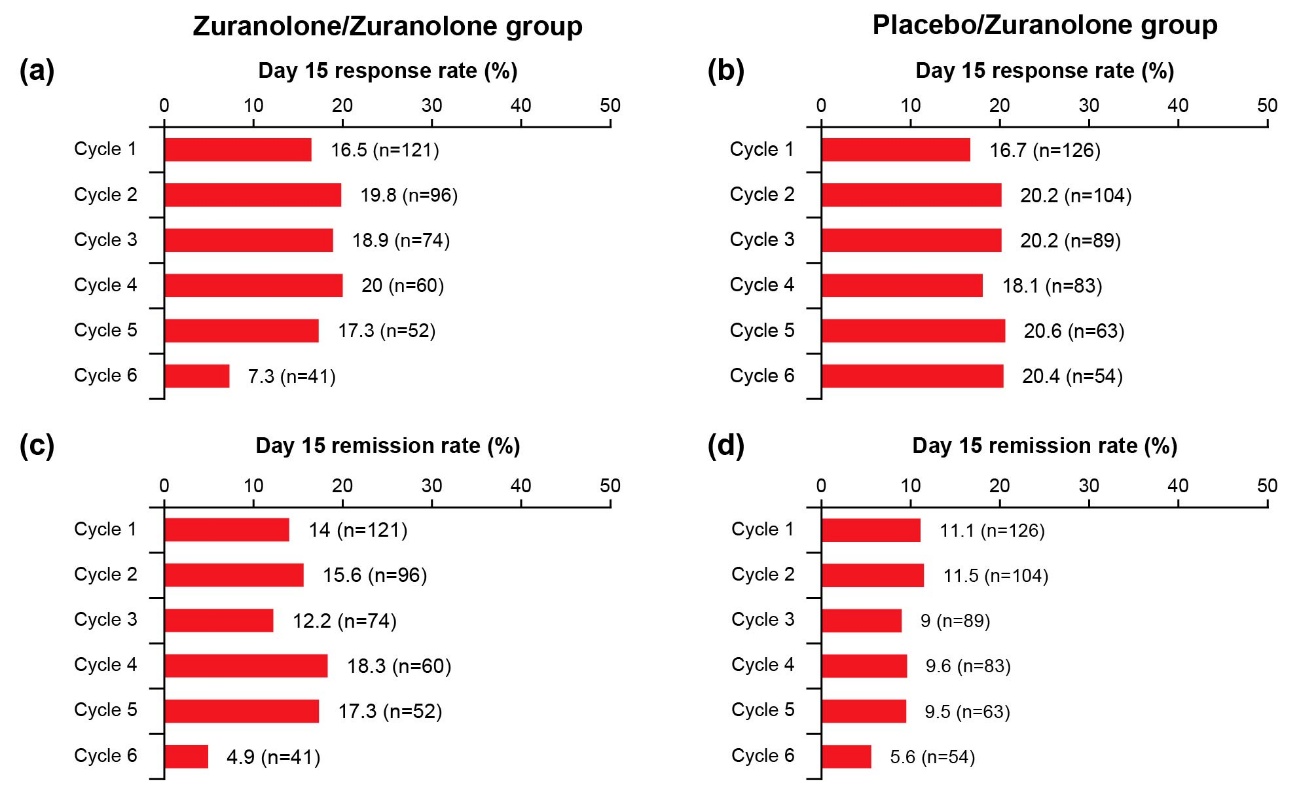


**Supplementary Fig. 5** Days to first HAMD-17 remission (cumulative rate of first remission) from their first treatment of zuranolone.


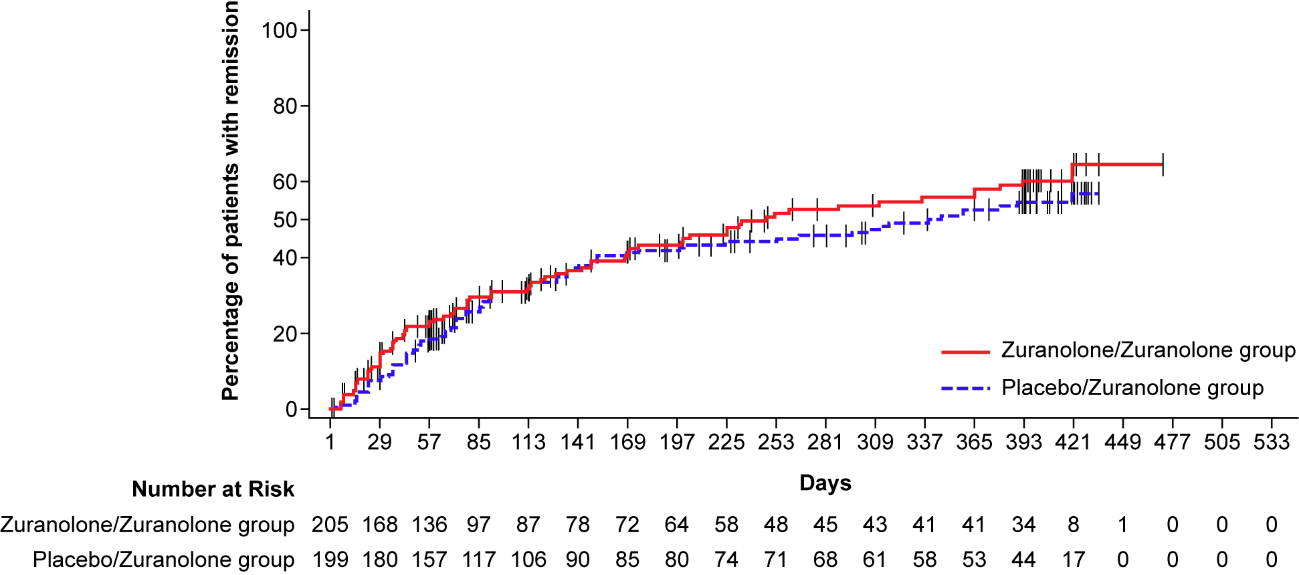


**Supplementary Fig. 6** Plasma zuranolone concentration in Cycle 1 and Cycle 2 of Part B.


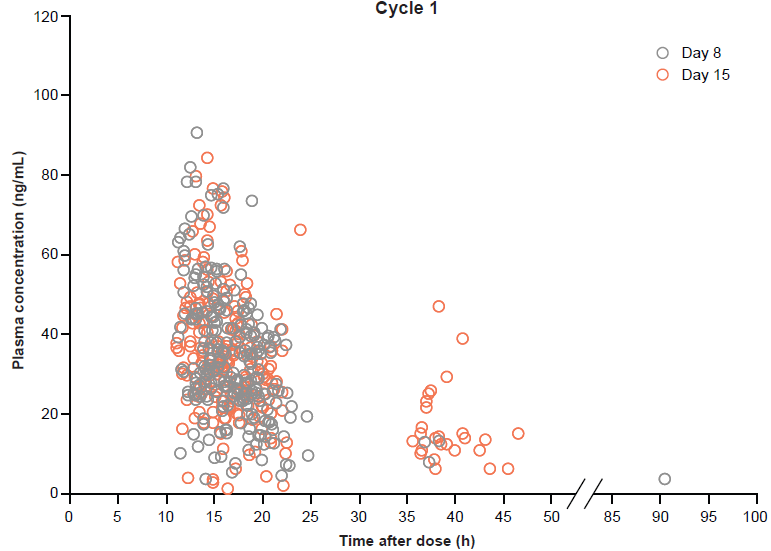


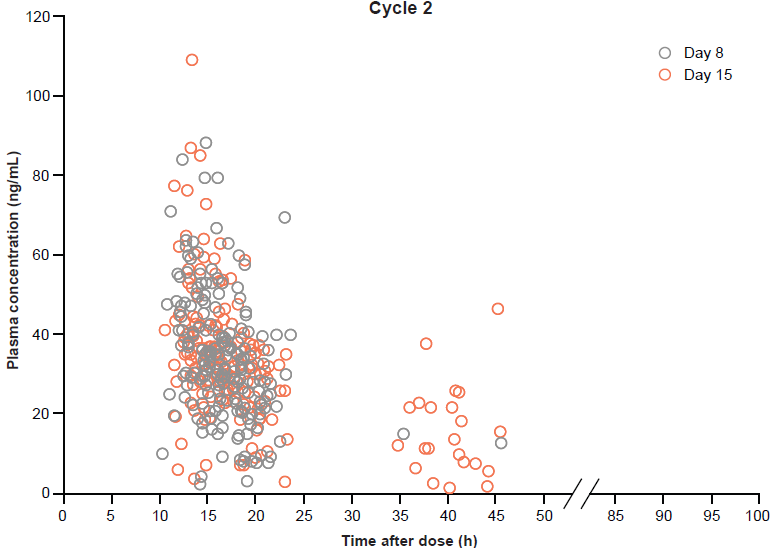


**Supplementary Table 1** Schedule of Activities in Part B

(a) Treatment period and follow-up period

| **Procedures** | | **Treatment Period** | | | **Follow-up Period** | | | | | | **Termination visit^f^ of treatment or follow-up period + 3 days** | **Follow-up visit^g^ after termination visit of treatment period**  **+ 3 days** |
| --- | --- | --- | --- | --- | --- | --- | --- | --- | --- | --- | --- | --- |
|  |  | **Visit 1**  **Day 1** | **Visit 2 Day 8 ± 1** | **Visit 3**  **Day 15 ± 1** | **Visit 4**  **Day 22 ± 2** | **Remote assessment^d^**  **Day 29 ± 2** | **Visit 5**  **Day 36 ± 2** | **Remote assessment^d^**  **Day 43 ± 2** | **Remote assessment^d^**  **Day 50 ± 2** | **Visit 6^e^ Day 57 ± 2** |  |  |
| JESS | | X |  |  |  |  |  |  |  |  |  |  |
| Physical examination | | X | X | X | X |  | X |  |  | X | X | X |
| Weight | | X |  | X | X |  | X |  |  | X | X | X |
| Pregnancy test^a^ | | X | X | X |  |  | X |  |  | X | X | X |
| Laboratory test^b^ | | X | X | X | X |  |  |  |  | X | X | X |
| Blood pressure, pulse rate | | X | X | X | X |  | X |  |  | X | X | X |
| 12-lead ECG | | X |  | X | X |  |  |  |  | X | X | X |
| Study intervention administration | | ←X^c^→ | | |  |  |  |  |  |  |  |  |
| HAMD-17 | | X | X | X | X |  | X |  |  | X | X |  |
| CGI-I | |  | X | X | X |  | X |  |  | X | X |  |
| CGI-S | | X | X | X | X |  | X |  |  | X | X |  |
| PGI-I (ePRO) | |  | X | X | X |  | X |  |  | X | X |  |
| PHQ-9^h^ (ePRO) | | X | X | X | X | X | X | X | X | X | X |  |
| SF-36 (pPRO) | | X |  | X |  |  | X |  |  |  | X |  |
| ISI (ePRO) | | X | X | X | X |  | X |  |  | X | X |  |
| Sleep diary (ePRO) | If a participant did not consent to the  weekly evaluation | ←X^i^→ | | | X^j^ |  | X^j^ |  |  | X^j^ | X^k^ |  |
|  | If a participant consented to the  weekly evaluation^r^ |  | | | X^s^ | X^s^ | X^s^ | X^s^ | X^s^ | X^s^ | X^l^ |  |
| Measurement of sleep/activity levels^r^ (Only for participants who consented to ePRO evaluation) | | ←X^m^→ | | | | | | | | | |  |
| Patient diary^n,r^ (only for participants who consented to ePRO evaluation) | | X | X | X | X | X | X | X | X | X | X |  |
| C-SSRS | | X | X | X | X |  | X |  |  | X | X | X |
| D-2-A | |  | X | X |  |  |  |  |  |  | X^o^ |  |
| D-2-B | |  |  |  | X |  | X |  |  | X | X^o^ |  |
| DEQ-5 (ePRO) | | X | X | X | X |  | X |  |  | X | X | X |
| AE review | | ←X→ | | | | | | | | | | |
| Concomitant medication therapy | | ←X→ | | | | | | | | | | |
| Blood sampling for PK | |  | X^p^ | X^p^ |  |  |  |  |  |  | X^q^ |  |

AE, adverse event; CGI-I, Clinical Global Impression - Global Improvement; CGI-S, Clinical Global Impression - Severity of Illness; C-SSRS, Columbia-Suicide Severity Rating Scale; D-2-A, Dependence-2A; D-2-B, Dependence-2B; DEQ-5, Drug Effect Questionnaire-5; ECG, electrocardiogram; ePRO, electronic patient-reported outcome;

HAMD-17, 17-item Hamilton Rating Scale for Depression; ISI, Insomnia Severity Index; JESS, Japanese version of the Epworth Sleepiness Scale; PGI-I, Patient Global Impression of Improvement; PHQ-9, Patient Health Questionnaire-9; PK, pharmacokinetics; pPRO, paper patient-reported outcome; SF-36, Short Form Health Survey

1. Only for women of childbearing potential.
2. Blood sampling for laboratory tests were performed in the fasted state (fasted for at least 10 hours) at Visit 1 (Day 1), at Visit 3 (Day 15 ± 1), and at the termination visit of the treatment period. At other visits as well, blood samples had to be collected in the fasted state to the extent possible.
3. Participants received the study intervention once daily on Day 1 to Day 14.
4. Remote assessment by telephone, etc. was performed.
5. Participants who met the Criteria for Starting the Treatment Period of Part B at Visit 6 (Day 57 ± 2) entered the treatment period within 1 week. At Visit 6 (Day 57 ± 2), participants who did not meet the criteria entered the durability observation period. The decision on whether a participant was to enter the treatment period or the durability observation period made within the acceptable time window for the final visit (Visit 6 [Day 57 ± 2]) in the follow-up period of Part B was based on the HAMD-17 total score and the duration of depressive episode as of the visit. For participants who entered the treatment period within the acceptable time window, the final visit (Visit 6

[Day 57 ± 2]) of the follow-up period of Part B could serve as the Visit 1 (Day 1) of the subsequent treatment period of Part B. For participants who entered the treatment period or the durability observation period outside of the acceptable time window for the final visit of the follow-up period, reassessments including HAMD-17 total score were performed to determine whether they were to enter the treatment period or the durability observation period.

1. To the extent possible, the termination visit was performed within 3 days from the day of termination. At the time point that 1 year (52 weeks) passed after the first dose in the last participant who agreed to participate in Part B, participants in the treatment period had to make a “termination visit of the treatment period” approximately 1 week after the end of the treatment period (Visit 1 to Visit 3 [Day 1 to 15 ± 1]), and those in the follow-up period had to make a “termination visit of the follow-up period” on an earliest possible occasion more than 1 week after the last dose as directed by the sponsor.
2. Follow-up visit after the termination visit of the treatment period was performed within 3 days from 1 week after the termination visit.
3. Whenever a participant gave 3 points to item 9 of PHQ-9 (thoughts that you would be better off dead, or of hurting yourself in some way), the investigator or subinvestigator (and the study coordinator if necessary) had to be alerted. The investigator or subinvestigator (or study coordinator) who received the alert had to contact the participant promptly to check participant’s the condition.
4. During Visit 1 to Visit 3 (Day 1 to Day 15 ± 1), participants evaluated their sleep every day from the bedtime on the day before to the awakening in the morning.
5. Participants evaluated their sleep from the bedtime on the day of visit to the awakening in the next morning.
6. Participants evaluated their sleep from the bedtime on the day before the visit to the awakening in the morning of the day of visit.
7. At the visit where the tablet computer was collected, participants evaluated their sleep from bedtime on the day before to the awakening in the morning of the day of visit.
8. To be performed daily from the beginning to the end of Part B.
9. Activities from 1 week before to the day before each time point were evaluated weekly.

o D-2-A was used at the termination visit of the treatment period. D-2-B was used at the termination visit of the follow-up period.

1. No blood sampling for PK was performed from the treatment cycle 3.
2. To be performed only if the participant discontinued during the treatment period.
3. To be performed only if the participant consented to the additional evaluation of sleep/activities (measurement of sleep/activity levels using the wearable device Fitbit Charge 4 [Fitbit], patient diary, and sleep diary with weekly evaluation).
4. To be evaluated weekly.

(b) Durability observation period

| Procedures | | Durability observation period^c^  Assessments for Week 1 to Week 8 were repeated every 8 weeks. | | | | | End of  study visit^d^  (± 7 days) | Termination visit^e^ of the durability  observation period |
| --- | --- | --- | --- | --- | --- | --- | --- | --- |
|  | | Remote  assessment  Week 1,3,5,7  (± 1 day) | Remote examination/  Telephone, etc. Week 2, 6 (± 2 days) | Remote examination/  Telemedicine  (or visit)  Week 4 (± 5 days) | Week 8 visit  (± 5 days) | Treatment  decision visit^o^ |  |  |
| Physical examination | |  | X^f^ | X^g^ | X | X | X | X |
| Weight | |  |  |  | X | X | X | X |
| Pregnancy test^a^ | |  |  |  | X | X | X | X |
| Laboratory test^b^ | |  |  |  | X | X | X | X |
| Blood pressure, pulse rate | |  |  |  | X | X | X | X |
| 12-lead ECG | |  |  |  | X | X | X | X |
| HAMD-17 | |  |  | X^g^ | X | X | X | X |
| CGI-I | |  |  |  | X | X | X | X |
| CGI-S | |  |  |  | X | X | X | X |
| PGI-I (ePRO) | |  |  |  | X | X | X | X |
| PHQ-9 (ePRO) | | X^h^ | X^h^ | X^h^ | X | X | X | X |
| ISI (ePRO) | |  |  |  | X | X | X | X |
| Sleep diary (ePRO) | If a participant did not consent to the weekly evaluation |  |  |  | X^i^ | X^i^ | X^j^ | X^j^ |
|  | If a participant consented to the weekly evaluation^n^ | X^p^ | X^p^ | X^p^ | X | X | X^k^ | X^k^ |
| Measurement of sleep/activity levels^n^ (Only for participants who consented to ePRO evaluation) | | ←X ^l^→ | | | | | | |
| Patient diary^m,^ ^n^ (only for participants who consented to ePRO evaluation) | | X | X | X | X | X | X | X |
| C-SSRS | |  |  |  | X | X | X | X |
| D-2-B | |  |  |  | X | X | X | X |
| DEQ-5 (ePRO) | |  |  |  | X | X | X | X |
| AE review | | ←X→ | | | | | | |
| Concomitant medication therapy | | ←X → | | | | | | |
| AE, adverse event; CGI-I, Clinical Global Impression - Global Improvement; CGI-S, Clinical Global Impression - Severity of Illness; C-SSRS, Columbia-Suicide Severity Rating Scale; D-2-B, Dependence-2B; DEQ-5, Drug Effect Questionnaire-5; ECG, electrocardiogram; ePRO, electronic patient-reported outcome; HAM-D17, 17-item Hamilton Rating Scale for Depression; ISI, Insomnia Severity Index; PGI-I, Patient Global Impression of Improvement; PHQ-9, Patient Health Questionnaire-9   1. Only for women of childbearing potential. 2. Blood sampling for laboratory tests was performed in the fasted state (fasted for at least 10 hours) at the termination visit of the durability observation period. At other visits as well, blood samples had to be collected in the fasted state to the extent possible. 3. For the durability observation period, Week 1 was defined as the day 1 week after the reference date, which was defined as the final visit (Visit 10 [Day 57 ± 2]) of Part A or the end of the follow-up period (Visit 6 [Day 57 ± 2]) in the last treatment cycle of Part B. Participants visited the study site to have the scheduled assessments every 8 weeks from the reference date of the durability observation period, which was defined as the final visit (Visit 10 [Day 57 ± 2]) of Part A or the end of the follow-up period (Visit 6 [Day 57 ± 2]) of the last treatment cycle of Part B. Participants who met the Criteria for Starting the Treatment Period of Part B on the visit entered the treatment period within 1 week. The decision that a participant was to enter the treatment period made within the acceptable time window for the treatment decision and Week 8 visit (± 5 days) was based on the HAMD-17 total score and the duration of depressive episode as of the visit. To enter the treatment period outside the acceptable time window for the specified visit, participants needed reassessments including HAMD-17 total score and had to meet the Criteria for Starting the Treatment Period in Part B. Participants who did not meet the Criteria for Starting the Treatment Period of Part B at Week 8 visit repeated the durability observation period. For participants who entered the treatment period in Part B on the basis of decision at the treatment decision visit or Week 8 visit, these visits could serve as the Visit 1 (Day 1) of the treatment visit of Part B. 4. Participants who entered the treatment period made the “end-of-study visit” 1 year (52 weeks) after the initial dose in Part B. Participants who did not enter the treatment   period made the “end-of-study visit” 1 year (52 weeks) after the start of study intervention in Part A.   1. To the extent possible, the termination visit was performed within 3 days from the day of termination. At the time point that 1 year (52 weeks) passed after the first dose in the last participant who agreed to participate in Part B, participants in the durability observation period had to make a “termination visit of the durability observation period” promptly as directed by the sponsor. 2. The examination was performed by telephone, etc. 3. Examination/assessment using the telemedicine application was also acceptable. If telemedicine was not feasible, the participant had to visit the study site for examination. 4. To be evaluated weekly. Whenever a participant’s rating of the PHQ-9 total score exceeded 10 or the physician saw the necessity of treatment, the participant had to return to the study site within 1 week for a treatment decision visit. If the Criteria for Starting the Treatment Period of Part B were met at this point, participants entered the treatment period within 1 week. 5. Participants evaluated their sleep from the bedtime on the day of visit to the awakening in the next morning. 6. Participants evaluated their sleep from the bedtime on the day before to the awakening in the morning of the day of visit. 7. At the visit where the tablet computer was collected, participants evaluated their sleep from bedtime on the day before to the awakening in the morning of the day of visit. 8. To be performed daily from the beginning to the end of Part B. 9. Activities from 1 week before to each time point were evaluated weekly. 10. To be performed only if the participant consented to the additional evaluation of sleep/activities (measurement of sleep/activity levels using the wearable device Fitbit Charge 4 [Fitbit], patient diary, and sleep diary with weekly evaluation). 11. Participants who met the Criteria for Starting the Treatment Period of Part B at the visit entered the treatment period on that day. Participants who could not do so had to enter the treatment period within 1 week. To enter the treatment period after the treatment decision visit + 5 days, participants had to undergo reassessments including HAMD-17 total score and meet the Criteria for Starting the Treatment Period in Part B. 12. To be evaluated weekly. | | | | | | | | |

**Supplementary Table 2** Details of study drug continuation, prohibited prior/concomitant therapy, restrictions on prior/concomitant therapy, general restrictions, and criteria for study drug discontinuation.

| **Continuation of study treatment after completion of the study**   - Administration of the investigational product is not planned after the completion of the clinical trial   **Prohibited Prior/Concomitant Therapy**   - - Participants had to abstain from taking the following drugs from the time of participation in Part B until the end or termination of Part B.   - Strong inhibitors of CYP3A   - Strong inducers of CYP3A   - Antidepressants   - Anxiolytics   - Hypnotics (excluding non- GABA hypnotics)   - Antipsychotics   - Antiparkinsonian drugs   - Antiepileptic drugs   - Mood stabilizers (lithium carbonate, carbamazepine, sodium valproate, lamotrigine, etc.)   - Other GABA_A_ receptor modulators or drugs that affect the GABA receptor or the amount of GABA   - Therapies using devices such as electroconvulsive therapy or transcranial   magnetic stimulation   - - Opioid preparations   **Restrictions on Prior/Concomitant Therapy**   - Use of the following medications was allowed on an as-needed basis up to twice a week from the time of participation in Part B until the end or termination of Part B.   However, the use on the day before each visit was prohibited.   - - Non-GABA type hypnotics (ramelteon, suvorexant, lemborexant, etc.)   - Drugs and Chinese herbal medicines used for the treatment of insomnia   (antihistamines, etc.) that are not listed above among the prohibited  concomitant medications  **Restrictions**   - Food and beverage containing grapefruits and other fruits - Participants had to abstain from grapefruit, Seville orange, and food or beverage containing these from 14 days prior to the start of each treatment period until the end of study intervention administration in the same treatment cycle. - Food and beverage containing alcohol - Participants had to abstain from food or beverage containing alcohol from the day before the start of each treatment period until 7 days after the end of study intervention administration in the same treatment cycle.   **Study drug discontinuation**   - The liver chemistry stopping criteria   Study drug administration was discontinued when any of the conditions in the below algorithm were met. Study drug discontinuation was also possible based on the liver function test and investigator’s discretion.  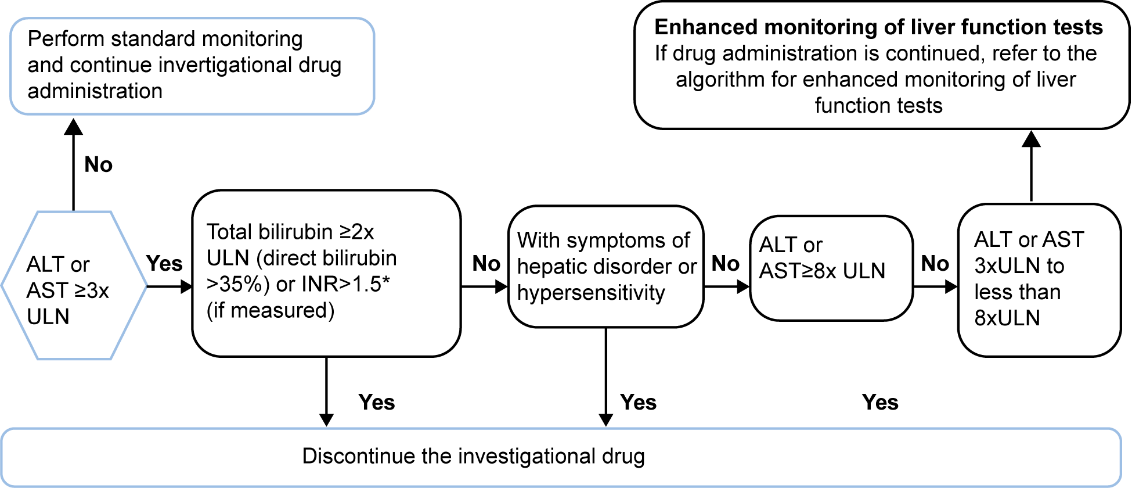   - The corrected QT (QTc) stopping criteria   Study drug administration was discontinued when the 12-lead ECG results (mean of triplicate measurements) met any of the conditions below:   - - QTcF >500 msec   - Change from baseline in QTcF >60 msec   - In case of bundle branch block, QTc >500 msec if baseline QTc was <450 msec; QTc ≥530 msec if baseline QTc was 450–480 msec - Cases of AEs - A participant’s request - An investigator or subinvestigator’s request - Participant’s pregnancy   Female patients who became pregnant during the study period discontinued the study drug treatment or were withdrawn from the study.   - Protocol deviation - A serious or intolerable AE occurred and the investigator or subinvestigator   considered that the participant had to be withdrawn   - The participant requested withdrawal - The participant was found to be ineligible for the study after the start of study   intervention   - The participant was lost to follow-up - The participant died - The investigator or subinvestigator considered that the target disease (depression)   required treatment other than that specified in the study protocol   - The investigator or subinvestigator determined that the participant had to be   withdrawn from the study for any other reasons |
| --- |

*****The INR criteria does not apply to subjects receiving anticoagulants.

**Supplementary Table 3** Demographics and baseline characteristics of participants in Cycle 1 in Part B by treatment assignment in Part A

| **Characteristics** | **Zuranolone/zuranolone group N = 128 n (%)** | **Placebo/zuranolone group N = 143 n (%)** |
| --- | --- | --- |
| Sex |  |  |
| Male | 64 (50.0) | 72 (50.3) |
| Female | 64 (50.0) | 71 (49.7) |
| Age, years |  |  |
| Mean (SD) | 39.7 (11.8) | 40.2 (12.1) |
| Median (range) | 39.0 (18-65) | 40.0 (18-68) |
| Age group, years |  |  |
| ≥ 18 to < 25 | 15 (11.7) | 14 (9.8) |
| ≥ 25 to < 45 | 68 (53.1) | 72 (50.3) |
| ≥ 45 to < 65 | 44 (34.4) | 55 (38.5) |
| ≥ 65 | 1 (0.8) | 2 (1.4) |
| BMI, kg/m^2^ |  |  |
| Mean (SD) | 23.3 (5.3) | 23.3 (4.0) |
| Race |  |  |
| Asian | 128 (100.0) | 143 (100.0) |
| HAMD-17 Total Score |  |  |
| Mean (SD) | 19.2 (3.7) | 19.4 (3.7) |
| Median (range) | 19.0 (14-31) | 19.0 (14-30) |
| ≤24 | 117 (91.4) | 128 (89.5) |
| ≥25 | 11 (8.6) | 15 (10.5) |
| PHQ-9 Total Score |  |  |
| Mean (SD) | 15.2 (4.9) | 15.7 (4.8) |
| Median (range) | 16.0 (1-25) | 15.0 (6-27) |
| DSM-5 classification |  |  |
| Single Episode | 48 (37.5) | 56 (39.2) |
| Recurrent | 80 (62.5) | 87 (60.8) |
| Episode recurrences |  |  |
| 1st time | 48 (37.5) | 56 (39.2) |
| 2nd time | 45 (35.2) | 51 (35.7) |
| 3rd-7th time | 34 (26.6) | 36 (25.2) |
| ≥8 times | 0 | 0 |
| Unknown | 1 (0.8) | 0 |
| Duration of current episode at randomization, months |  |  |
| Mean (SD) | 5.94 (2.97) | 5.83 (2.77) |
| Median (range) | 5.21 (2.3-12.4) | 5.19 (2.2-12.0) |
| 2-4 | 47 (36.7) | 53 (37.1) |
| 4-6 | 29 (22.7) | 32 (22.4) |
| 6-8 | 19 (14.8) | 26 (18.2) |
| 8-10 | 12 (9.4) | 15 (10.5) |
| 10-12 | 18 (14.1) | 17 (11.9) |
| ≥12 | 3 (2.3) | 0 |
| Prior drug therapies for depressive episodes |  |  |
| Yes | 70 (54.7) | 80 (55.9) |
| No | 58 (45.3) | 63 (44.1) |
| Previous disease | 11 (8.6) | 15 (10.5) |
| Concurrent disease | 93 (72.7) | 101 (70.6) |
| Employment status |  |  |
| Full-time (≥35 hours per week) | 43 (33.6) | 56 (39.2) |
| Part-time (<35 hours per week) | 20 (15.6) | 17 (11.9) |
| Unemployed | 36 (28.1) | 24 (16.8) |
| Retired | 5 (3.9) | 13 (9.1) |
| Other | 24 (18.8) | 33 (23.1) |

BMI, body mass index; DSM-5, Diagnostic and Statistical Manual of Mental Disorders, 5th edition; HAMD-17, 17-item Hamilton Rating Scale for Depression; Max, maximum; Min, minimum; PHQ-9, Patient Health Questionnaire-9; SD, standard deviation

**Supplementary Table 4** TEAEs by total treatment cycles in Part B (Safety Set).

|  | **Zuranolone/zuranolone group** | | | | | | **Placebo/zuranolone** | | | | | |
| --- | --- | --- | --- | --- | --- | --- | --- | --- | --- | --- | --- | --- |
|  | Cycle 1 | Cycle 2 | Cycle 3 | Cycle 4 | Cycle 5 | Cycle 6 | Cycle 1 | Cycle 2 | Cycle 3 | Cycle 4 | Cycle 5 | Cycle 6 |
|  | (n=128) | (n=99) | (n=76) | (n=63) | (n=55) | (n=43) | (n=143) | (n=114) | (n=94) | (n=84) | (n=71) | (n=56) |
| **Treatment period** |  |  |  |  |  |  |  |  |  |  |  |  |
| Participants with any TEAE, n (%) | 51 (39.8) | 29 (29.3) | 19 (25.0) | 19 (30.2) | 13 (23.6) | 7 (16.3) | 53 (37.1) | 30 (26.3) | 21 (22.3) | 16 (19.0) | 18 (25.4) | 8 (14.3) |
| Severe | 1 (0.8) | 0 | 0 | 0 | 0 | 0 | 0 | 2 (1.8) | 0 | 0 | 0 | 0 |
| Moderate | 6 (4.7) | 3 (3.0) | 3 (3.9) | 3 (4.8) | 2 (3.6) | 0 | 14 (9.8) | 3 (2.6) | 3 (3.2) | 5 (6.0) | 0 | 2 (3.6) |
| Mild | 44 (34.4) | 26 (26.3) | 16 (21.1) | 16 (25.4) | 11 (20.0) | 7 (16.3) | 39 (27.3) | 25 (21.9) | 18 (19.1) | 11 (13.1) | 18 (25.4) | 6 (10.7) |
| TEAE in ≥5% of subjects at any time, n (%) |  |  |  |  |  |  |  |  |  |  |  |  |
| - Somnolence | 14 (10.9) | 8 (8.1) | 2 (2.6) | 2 (3.2) | 2 (3.6) | 0 | 19 (13.3) | 4 (3.5) | 2 (2.1) | 2 (2.4) | 1 (1.4) | 2 (3.6) |
| - Dizziness | 13 (10.2) | 4 (4.0) | 3 (3.9) | 2 (3.2) | 2 (3.6) | 2 (4.7) | 10 (7.0) | 3 (2.6) | 3 (3.2) | 3 (3.6) | 4 (5.6) | 1 (1.8) |
| - Feeling abnormal | 4 (3.1) | 3 (3.0) | 3 (3.9) | 4 (6.3) | 3 (5.5) | 2 (4.7) | 4 (2.8) | 3 (2.6) | 1 (1.1) | 0 | 1 (1.4) | 0 |
| - Asthenia | 2 (1.6) | 2 (2.0) | 2 (2.6) | 2 (3.2) | 3 (5.5) | 1 (2.3) | 1 (0.7) | 1 (0.9) | 1 (1.1) | 0 | 0 | 0 |
| **Follow-up period** |  |  |  |  |  |  |  |  |  |  |  |  |
| Participants with any TEAE, n (%) | 33 (25.8) | 15 (15.2) | 17 (22.4) | 8 (12.7) | 9 (16.4) | 9 (20.9) | 33 (23.1) | 28 (24.6) | 17 (18.1) | 12 (14.3) | 7 (9.9) | 15 (26.8) |
| Severe | 0 | 0 | 0 | 0 | 0 | 0 | 0 | 1 (0.9) | 0 | 0 | 1 (1.4) | 0 |
| Moderate | 7 (5.5) | 5 (5.1) | 5 (6.6) | 2 (3.2) | 3 (5.5) | 3 (7.0) | 10 (7.0) | 6 (5.3) | 4 (4.3) | 4 (4.8) | 3 (4.2) | 5 (8.9) |
| Mild | 26 (20.3) | 10 (10.1) | 12 (15.8) | 6 (9.5) | 6 (10.9) | 6 (14.0) | 23 (16.1) | 21 (18.4) | 13 (13.8) | 8 (9.5) | 3 (4.2) | 10 (17.9) |
| TEAE in ≥ 5% of subjects at any time, n (%) |  |  |  |  |  |  |  |  |  |  |  |  |
| Nasopharyngitis | 6 (4.7) | 4 (4.0) | 3 (3.9) | 0 | 2 (3.6) | 1 (2.3) | 3 (2.1) | 2 (1.8) | 4 (4.3) | 0 | 0 | 6 (10.7) |

TEAE, treatment-emergent adverse event

**Supplementary Table 5** Incidence of TEAEs stratified by sex

|  | **Male**  **(n=98)**  **n (%)** | **Female**  **(n=101)**  **n (%)** |
| --- | --- | --- |
| **Participants with any TEAE** | 40 (40.8) | 46 (45.5) |
| Infections and infestations | 14 (14.3) | 13 (12.9) |
| - COVID-19 | 8 (8.2) | 4 (4.0) |
| - Nasopharyngitis | 6 (6.1) | 5 (5.0) |
| - Urinary tract infection | 0 | 4 (4.0) |
| - Bacteriuria | 0 | 1 (1.0) |
| - Herpes virus infection | 0 | 1 (1.0) |
| - Influenza | 1 (1.0) | 0 |
| - Pharyngitis | 0 | 1 (1.0) |
| - Rhinitis | 1 (1.0) | 0 |
| - Sinusitis | 1 (1.0) | 0 |
| Metabolism and nutrition disorders | 2 (2.0) | 1 (1.0) |
| - Gout | 1 (1.0) | 0 |
| - Hyperuricaemia | 1 (1.0) | 0 |
| - Increased appetite | 0 | 1 (1.0) |
| Psychiatric disorders | 2 (2.0) | 1 (1.0) |
| - Insomnia | 1 (1.0) | 1 (1.0) |
| - Nightmare | 1 (1.0) | 0 |
| Nervous system disorders | 7 (7.1) | 10 (9.9) |
| - Somnolence | 5 (5.1) | 7 (6.9) |
| - Headache | 1 (1.0) | 3 (3.0) |
| - Dizziness | 1 (1.0) | 2 (2.0) |
| - Head discomfort | 1 (1.0) | 0 |
| Eye disorders | 1 (1.0) | 2 (2.0) |
| - Cataract | 0 | 1 (1.0) |
| - Conjunctivitis allergic | 0 | 1 (1.0) |
| - Vitreous floaters | 1 (1.0) | 0 |
| Ear and labyrinth disorders | 0 | 1 (1.0) |
| - Tinnitus | 0 | 1 (1.0) |
| Cardiac disorders | 2 (2.0) | 1 (1.0) |
| - Bradycardia | 0 | 1 (1.0) |
| - Supraventricular extrasystoles | 1 (1.0) | 0 |
| - Ventricular extrasystoles | 1 (1.0) | 0 |
| Vascular disorders | 1 (1.0) | 0 |
| - Hypertension | 1 (1.0) | 0 |
| Respiratory, thoracic and mediastinal disorders | 2 (2.0) | 2 (2.0) |
| - Cough | 2 (2.0) | 1 (1.0) |
| - Rhinitis allergic | 0 | 1 (1.0) |
| Gastrointestinal disorders | 5 (5.1) | 14 (13.9) |
| - Diarrhoea | 3 (3.1) | 3 (3.0) |
| - Constipation | 1 (1.0) | 4 (4.0) |
| - Abdominal pain upper | 0 | 3 (3.0) |
| - Abdominal discomfort | 0 | 2 (2.0) |
| - Nausea | 0 | 2 (2.0) |
| - Abdominal distension | 0 | 1 (1.0) |
| - Abdominal pain | 0 | 1 (1.0) |
| - Dental caries | 1 (1.0) | 0 |
| - Haemorrhoids | 1 (1.0) | 0 |
| - Periodontal disease | 0 | 1 (1.0) |
| - Vomiting | 0 | 1 (1.0) |
| - Anal haemorrhage | 0 | 1 (1.0) |
| - Large intestine polyp | 1 (1.0) | 0 |
| Hepatobiliary disorders | 1 (1.0) | 0 |
| - Hepatic function abnormal | 1 (1.0) | 0 |
| Skin and subcutaneous tissue disorders | 3 (3.1) | 4 (4.0) |
| - Pruritus | 0 | 2 (2.0) |
| - Dermatitis | 1 (1.0) | 0 |
| - Dry skin | 1 (1.0) | 0 |
| - Eczema | 0 | 1 (1.0) |
| - Rash | 0 | 1 (1.0) |
| - Urticaria | 1 (1.0) | 0 |
| Musculoskeletal and connective tissue disorders | 4 (4.1) | 2 (2.0) |
| - Back pain | 2 (2.0) | 1 (1.0) |
| - Myalgia | 1 (1.0) | 0 |
| - Temporomandibular joint syndrome | 0 | 1 (1.0) |
| - Fibromyalgia | 1 (1.0) | 0 |
| Renal and urinary disorders | 0 | 1 (1.0) |
| - Pollakiuria | 0 | 1 (1.0) |
| General disorders and administration site conditions | 4 (4.1) | 6 (5.9) |
| - Pyrexia | 1 (1.0) | 3 (3.0) |
| - Malaise | 1 (1.0) | 2 (2.0) |
| - Chest pain | 0 | 1 (1.0) |
| - Feeling abnormal | 1 (1.0) | 0 |
| - Injection site bruising | 1 (1.0) | 0 |
| - Injection site pain | 0 | 1 (1.0) |
| - Pain | 0 | 1 (1.0) |
| Investigations | 10 (10.2) | 3 (3.0) |
| - Alanine aminotransferase increased | 3 (3.1) | 0 |
| - Urinary occult blood positive | 3 (3.1) | 0 |
| - Blood uric acid increased | 2 (2.0) | 0 |
| - Aspartate aminotransferase increased | 1 (1.0) | 0 |
| - Blood creatine phosphokinase increased | 0 | 1 (1.0) |
| - Blood glucose increased | 1 (1.0) | 0 |
| - Glucose urine present | 0 | 1 (1.0) |
| - Weight decreased | 1 (1.0) | 0 |
| - Weight increased | 1 (1.0) | 0 |
| - Urine leukocyte esterase positive | 0 | 1 (1.0) |
| Injury, poisoning and procedural complications | 6 (6.1) | 2 (2.0) |
| - Ligament sprain | 2 (2.0) | 0 |
| - Arthropod sting | 1 (1.0) | 0 |
| - Vaccination complication | 1 (1.0) | 0 |
| - Wound | 0 | 1 (1.0) |
| - Crush injury | 1 (1.0) | 0 |
| - Heat illness | 1 (1.0) | 0 |
| - Post-traumatic neck syndrome | 1 (1.0) | 0 |
| - Tooth dislocation | 0 | 1 (1.0) |
| - Palate injury | 1 (1.0) | 0 |

COVID-19, coronavirus disease off 2019; TEAE, treatment-emergent adverse event

**Supplementary Table 6** Mean change from baseline in HAMD-17 in Part B of the study in the full analysis set

|  |  | Baseline | Day 8 | Day 15 | Day 22 | Day 36 | Day 57 |
| --- | --- | --- | --- | --- | --- | --- | --- |
| Cycle 1 | n | 271 | 263 | 247 | 239 | 240 | 240 |
|  | Observed value  Mean (SD) | 19.3 (3.7) | 15.3 (4.9) | 14.1 (5.3) | 14.2 (5.5) | 14.8 (5.8) | 16.1 (5.7) |
|  | Change from baseline  Mean (SD) | - | -4.0 (4.1) | -5.0 (4.8) | -5.0 (4.7) | -4.4 (4.9) | -3.2 (4.9) |
| Cycle 2 | n | 213 | 206 | 200 | 201 | 199 | 190 |
|  | Observed value  Mean (SD) | 18.6 (3.3) | 14.7 (4.6) | 13.5 (5.6) | 13.8 (5.9) | 14.4 (5.8) | 15.8 (5.5) |
|  | Change from baseline  Mean (SD) | - | -3.9 (3.9) | -5.2 (4.8) | -4.9 (4.9) | -4.3 (5.0) | -2.9 (4.5) |
| Cycle 3 | n | 170 | 160 | 163 | 160 | 161 | 156 |
|  | Observed value  Mean (SD) | 18.0 (3.4) | 14.2 (4.5) | 13.3 (4.8) | 13.9 (4.9) | 14.4 (5.3) | 16.0 (4.9) |
|  | Change from baseline  Mean (SD) | - | -3.7 (3.8) | -4.6 (4.4) | -4.0 (4.2) | -3.5 (4.5) | -2.1 (4.4) |
| Cycle 4 | n | 147 | 142 | 143 | 141 | 139 | 137 |
|  | Observed value  Mean (SD) | 18.0 (3.2) | 13.9 (4.6) | 13.6 (5.0) | 14.0 (5.0) | 14.7 (5.3) | 16.4 (4.8) |
|  | Change from baseline  Mean (SD) | - | -4.1 (4.1) | -4.4 (4.6) | -4.0 (4.6) | -3.4 (4.7) | -1.7 (4.3) |
| Cycle 5 | n | 126 | 122 | 115 | 118 | 108 | 102 |
|  | Observed value  Mean (SD) | 18.1 (3.2) | 14.1 (4.7) | 13.6 (5.2) | 14.1 (5.5) | 15.5 (4.8) | 17.1 (4.6) |
|  | Change from baseline  Mean (SD) | - | -4.0 (4.1) | -4.6 (4.8) | -4.0 (5.0) | -2.6 (4.3) | -1.2 (4.3) |
| Cycle 6 | n | 99 | 91 | 95 | 95 | 92 | 85 |
|  | Observed value  Mean (SD) | 18.2 (3.2) | 14.6 (4.5) | 14.0 (4.9) | 14.5 (5.2) | 15.3 (5.5) | 15.5 (5.6) |
|  | Change from baseline  Mean (SD) | - | -3.6 (3.8) | -4.1 (4.2) | -3.7 (4.0) | -2.9 (4.3) | -2.9 (4.2) |

HAMD-17, 17-item Hamilton Depression Rating Scale; SD, standard deviation

**Supplementary Table 7** Number of treatment cycles of zuranolone in Part B for participants with one-year follow-up from their first treatment of zuranolone.

| **Participants with one-year follow-up from their first treatment of zuranolone** | | | | | |
| --- | --- | --- | --- | --- | --- |
|  | | | **Zuranolone/zuranolone group (n = 95) n (%)** | **Placebo/zuranolone group (n = 89) n (%)** | **Total (n = 184)**  **n (%)** |
| Number of treatment cycle for zuranolone in Part B | 1 | | 11 (11.6) | 6 (6.7) | 17 (9.2) |
|  | 2 | | 10 (10.5) | 5 (5.6) | 15 (8.2) |
|  | 3 | | 8 (8.4) | 7 (7.9) | 15 (8.2) |
|  | 4 | | 6 (6.3) | 9 (10.1) | 15 (8.2) |
|  | 5 | | 10 (10.5) | 8 (9.0) | 18 (9.8) |
|  | 6 | | 37 (38.9) | 54 (60.7) | 91 (49.5) |
| **Participants with a response on day 15 of the first treatment of zuranolone*** | | | | | |
|  | | **Zuranolone/zuranolone group (n = 26) n (%)** | | **Placebo/zuranolone group (n = 15) n (%)** | **Total (n = 41)**  **n (%)** |
| Number of treatment cycle for zuranolone in Part B | 1 | 5 (19.2) | | 2 (13.3) | 7 (17.1) |
|  | 2 | 4 (15.4) | | 1 (6.7) | 5 (12.2) |
|  | 3 | 1 (3.8) | | 2 (13.3) | 3 (7.3) |
|  | 4 | 3 (11.5) | | 0 | 3 (7.3) |
|  | 5 | 2 (7.7) | | 4 (26.7) | 6 (14.6) |
|  | 6 | 5 (19.2) | | 6 (40.0) | 11 (26.8) |

*Participants who had assigned to zuranolone group in Part A and showed response with zuranolone in Part A or participants who had assigned to the placebo group in Part A and showed response with zuranolone in treatment cycle1 of Part B

**Supplementary Table 8** Rate of improvement by CGI-I at each timepoint in Part B

| Treatment cycles | Time Point | n | Number of Improvement | Improvement Rate (%) |  |
| --- | --- | --- | --- | --- | --- |
| Cycle 1 | Day 8 | 263 | 51 | 19.4 |  |
|  | Day 15 | 247 | 71 | 28.7 |  |
|  | Day 22 | 239 | 66 | 27.6 |  |
|  | Day 36 | 240 | 56 | 23.3 |  |
|  | Day 57 | 240 | 42 | 17.5 |  |
|  | Last observation | 271 | 28 | 10.3 |  |
| Cycle 2 | Day 8 | 206 | 35 | 17.0 |  |
|  | Day 15 | 200 | 59 | 29.5 |  |
|  | Day 22 | 201 | 64 | 31.8 |  |
|  | Day 36 | 199 | 56 | 28.1 |  |
|  | Day 57 | 190 | 34 | 17.9 |  |
|  | Last observation | 213 | 19 | 8.9 |  |
| Cycle 3 | Day 8 | 160 | 36 | 22.5 |  |
|  | Day 15 | 163 | 52 | 31.9 |  |
|  | Day 22 | 160 | 40 | 25.0 |  |
|  | Day 36 | 161 | 40 | 24.8 |  |
|  | Day 57 | 156 | 27 | 17.3 |  |
|  | Last observation | 169 | 13 | 7.7 |  |
| Cycle 4 | Day 8 | 142 | 35 | 24.6 |  |
|  | Day 15 | 144 | 39 | 27.1 |  |
|  | Day 22 | 141 | 30 | 21.3 |  |
|  | Day 36 | 139 | 28 | 20.1 |  |
|  | Day 57 | 137 | 17 | 12.4 |  |
|  | Last observation | 147 | 8 | 5.4 |  |
| Cycle 5 | Day 8 | 122 | 30 | 24.6 |  |
|  | Day 15 | 115 | 39 | 33.9 |  |
|  | Day 22 | 118 | 29 | 24.6 |  |
|  | Day 36 | 108 | 14 | 13.0 |  |
|  | Day 57 | 102 | 10 | 9.8 |  |
|  | Last observation | 126 | 19 | 15.1 |  |
| Cycle 6 | Day 8 | 91 | 18 | 19.8 |  |
|  | Day 15 | 95 | 28 | 29.5 |  |
|  | Day 22 | 95 | 22 | 23.2 |  |
|  | Day 36 | 92 | 16 | 17.4 |  |
|  | Day 57 | 85 | 17 | 20.0 |  |
|  | Last observation | 99 | 20 | 20.2 |  |
| CGI-I, Clinical Global Impression - Global Improvement | | | | | |
| "Improvement" is defined as rating of "Very much improved" or "Much improved." | | | | | |

**Supplementary Table 9.** Summary statistics for ISI Total Score at each timepoint in Part B

| **Treatment cycles** | **Time point** | **Statistic** | **Total   N = 304** |  |
| --- | --- | --- | --- | --- |
| Cycle 1 | Baseline | n | 271 |  |
|  |  | Mean (SD) | 15.9 (5.8) |  |
|  | Day 8 | n | 268 |  |
|  |  | Mean (SD) | 13.0 (6.0) |  |
|  | - Change from Baseline | n | 268 |  |
|  |  | Mean (SD) | -3.0 (4.3) |  |
|  | Day 15 | n | 261 |  |
|  |  | Mean (SD) | 12.0 (6.3) |  |
|  | - Change from Baseline | n | 261 |  |
|  |  | Mean (SD) | -4.0 (5.4) |  |
|  | Day 22 | n | 253 |  |
|  |  | Mean (SD) | 12.9 (6.2) |  |
|  | - Change from Baseline | n | 253 |  |
|  |  | Mean (SD) | -3.1 (4.7) |  |
|  | Day 36 | n | 249 |  |
|  |  | Mean (SD) | 13.7 (6.4) |  |
|  | - Change from Baseline | n | 249 |  |
|  |  | Mean (SD) | -2.3 (4.2) |  |
|  | Day 57 | n | 243 |  |
|  |  | Mean (SD) | 14.8 (6.3) |  |
|  | - Change from Baseline | n | 243 |  |
|  |  | Mean (SD) | -1.2 (4.2) |  |
|  | Last observation | n | 271 |  |
|  |  | Mean (SD) | 15.2 (6.1) |  |
|  | - Change from Baseline | n | 271 |  |
|  |  | Mean (SD) | -0.8 (4.2) |  |
| Cycle 2 | Baseline | n | 213 |  |
|  |  | Mean (SD) | 16.2 (5.4) |  |
|  | Day 8 | n | 212 |  |
|  |  | Mean (SD) | 12.8 (5.9) |  |
|  | - Change from Baseline | n | 212 |  |
|  |  | Mean (SD) | -3.4 (4.6) |  |
|  | Day 15 | n | 208 |  |
|  |  | Mean (SD) | 12.2 (6.3) |  |
|  | - Change from Baseline | n | 208 |  |
|  |  | Mean (SD) | -4.1 (5.0) |  |
|  | Day 22 | n | 207 |  |
|  |  | Mean (SD) | 13.5 (6.2) |  |
|  | - Change from Baseline | n | 207 |  |
|  |  | Mean (SD) | -2.7 (4.6) |  |
|  | Day 36 | n | 204 |  |
|  |  | Mean (SD) | 14.1 (6.1) |  |
|  | - Change from Baseline | n | 204 |  |
|  |  | Mean (SD) | -2.1 (4.2) |  |
|  | Day 57 | n | 195 |  |
|  |  | Mean (SD) | 14.8 (6.2) |  |
|  | - Change from Baseline | n | 195 |  |
|  |  | Mean (SD) | -1.4 (4.0) |  |
|  | Last observation | n | 213 |  |
|  |  | Mean (SD) | 15.2 (5.9) |  |
|  | - Change from Baseline | n | 213 |  |
|  |  | Mean (SD) | -1.0 (4.1) |  |
| Cycle 3 | Baseline | n | 170 |  |
|  |  | Mean (SD) | 16.2 (5.3) |  |
|  | Day 8 | n | 170 |  |
|  |  | Mean (SD) | 12.6 (6.2) |  |
|  | - Change from Baseline | n | 170 |  |
|  |  | Mean (SD) | -3.6 (4.8) |  |
|  | Day 15 | n | 170 |  |
|  |  | Mean (SD) | 12.2 (6.3) |  |
|  | - Change from Baseline | n | 170 |  |
|  |  | Mean (SD) | -4.0 (5.3) |  |
|  | Day 22 | n | 165 |  |
|  |  | Mean (SD) | 13.7 (6.1) |  |
|  | - Change from Baseline | n | 165 |  |
|  |  | Mean (SD) | -2.5 (4.2) |  |
|  | Day 36 | n | 165 |  |
|  |  | Mean (SD) | 14.4 (6.1) |  |
|  | - Change from Baseline | n | 165 |  |
|  |  | Mean (SD) | -1.8 (4.0) |  |
|  | Day 57 | n | 159 |  |
|  |  | Mean (SD) | 15.3 (6.1) |  |
|  | - Change from Baseline | n | 159 |  |
|  |  | Mean (SD) | -0.9 (3.9) |  |
|  | Last observation | n | 170 |  |
|  |  | Mean (SD) | 15.3 (6.3) |  |
|  | - Change from Baseline | n | 170 |  |
|  |  | Mean (SD) | -0.9 (3.9) |  |
| Cycle 4 | Baseline | n | 147 |  |
|  |  | Mean (SD) | 16.2 (5.7) |  |
|  | Day 8 | n | 146 |  |
|  |  | Mean (SD) | 12.6 (6.3) |  |
|  | - Change from Baseline | n | 146 |  |
|  |  | Mean (SD) | -3.6 (5.2) |  |
|  | Day 15 | n | 145 |  |
|  |  | Mean (SD) | 12.5 (6.2) |  |
|  | - Change from Baseline | n | 145 |  |
|  |  | Mean (SD) | -3.7 (5.5) |  |
|  | Day 22 | n | 144 |  |
|  |  | Mean (SD) | 14.0 (5.8) |  |
|  | - Change from Baseline | n | 144 |  |
|  |  | Mean (SD) | -2.2 (4.6) |  |
|  | Day 36 | n | 138 |  |
|  |  | Mean (SD) | 14.6 (6.0) |  |
|  | - Change from Baseline | n | 138 |  |
|  |  | Mean (SD) | -1.8 (4.1) |  |
|  | Day 57 | n | 136 |  |
|  |  | Mean (SD) | 15.4 (5.9) |  |
|  | - Change from Baseline | n | 136 |  |
|  |  | Mean (SD) | -0.9 (3.6) |  |
|  | Last observation | n | 147 |  |
|  |  | Mean (SD) | 16.0 (5.7) |  |
|  | - Change from Baseline | n | 147 |  |
|  |  | Mean (SD) | -0.2 (3.6) |  |
| Cycle 5 | Baseline | n | 126 |  |
|  |  | Mean (SD) | 16.5 (5.6) |  |
|  | Day 8 | n | 125 |  |
|  |  | Mean (SD) | 13.0 (6.5) |  |
|  | - Change from Baseline | n | 125 |  |
|  |  | Mean (SD) | -3.5 (5.0) |  |
|  | Day 15 | n | 121 |  |
|  |  | Mean (SD) | 12.5 (6.6) |  |
|  | - Change from Baseline | n | 121 |  |
|  |  | Mean (SD) | -4.0 (5.4) |  |
|  | Day 22 | n | 123 |  |
|  |  | Mean (SD) | 14.1 (6.1) |  |
|  | - Change from Baseline | n | 123 |  |
|  |  | Mean (SD) | -2.4 (4.5) |  |
|  | Day 36 | n | 111 |  |
|  |  | Mean (SD) | 15.2 (5.7) |  |
|  | - Change from Baseline | n | 111 |  |
|  |  | Mean (SD) | -1.3 (4.0) |  |
|  | Day 57 | n | 105 |  |
|  |  | Mean (SD) | 16.1 (5.7) |  |
|  | - Change from Baseline | n | 105 |  |
|  |  | Mean (SD) | -0.4 (3.1) |  |
|  | Last observation | n | 126 |  |
|  |  | Mean (SD) | 15.4 (6.3) |  |
|  | - Change from Baseline | n | 126 |  |
|  |  | Mean (SD) | -1.1 (4.6) |  |
| Cycle 6 | Baseline | n | 99 |  |
|  |  | Mean (SD) | 16.7 (5.7) |  |
|  | Day 8 | n | 99 |  |
|  |  | Mean (SD) | 13.5 (6.3) |  |
|  | - Change from Baseline | n | 99 |  |
|  |  | Mean (SD) | -3.3 (4.9) |  |
|  | Day 15 | n | 99 |  |
|  |  | Mean (SD) | 13.2 (6.2) |  |
|  | - Change from Baseline | n | 99 |  |
|  |  | Mean (SD) | -3.5 (5.3) |  |
|  | Day 22 | n | 98 |  |
|  |  | Mean (SD) | 14.3 (6.4) |  |
|  | - Change from Baseline | n | 98 |  |
|  |  | Mean (SD) | -2.5 (4.3) |  |
|  | Day 36 | n | 92 |  |
|  |  | Mean (SD) | 14.8 (6.4) |  |
|  | - Change from Baseline | n | 92 |  |
|  |  | Mean (SD) | -2.2 (4.7) |  |
|  | Day 57 | n | 87 |  |
|  |  | Mean (SD) | 14.8 (6.4) |  |
|  | - Change from Baseline | n | 87 |  |
|  |  | Mean (SD) | -2.0 (4.8) |  |
|  | Last observation | n | 99 |  |
|  |  | Mean (SD) | 14.8 (6.6) |  |
|  | - Change from Baseline | n | 99 |  |
|  |  | Mean (SD) | -2.0 (4.7) |  |
| Max, maximum; Min, minimum; SD, standard deviation; ISI, Insomnia Severity Index | | | | |
